# Identification of kidney renal clear cell carcinoma prognosis based on gene expression and clinical information

**DOI:** 10.3389/fmolb.2025.1630250

**Published:** 2025-08-20

**Authors:** Xiong Zou, Xi Chen, Jianjun Yang, Yanfeng Li

**Affiliations:** Department of Urology, The Affiliated Hospital of Guizhou Medical University, Guiyang, Guizhou, China

**Keywords:** kidney renal clear cell carcinoma (KIRC), immune subtypes, risk stratification, immune microenvironment, prognosis

## Abstract

**Background:**

Kidney renal clear cell carcinoma (KIRC) prognosis exhibits substantial heterogeneity even among patients with identical clinicopathological staging, reflecting the limitations of current classification systems. Therefore, the development of reliable prognostic tools may improve clinical evaluation of KIRC outcomes and facilitate personalized therapy optimization.

**Methods:**

The KIRC data of GSE40435 and GSE46699 in the GEO database were immunologically grouped based on 29 immune gene sets through R language. At the same time, RNA sequencing data, clinical information and tumor mutation data of KIRC patients in the TCGA database were jointly processed to explore methods that facilitate clinicians to judge the prognosis of KIRC patients. Quantitative real-time PCR (qPCR) was performed to validate the expression of key prognostic related genes (PRGs) in KIRC and paired adjacent normal tissues.

**Results:**

There were significant differences in the immune microenvironment and genetic composition of different immune subtypes of KIRC. A number of high-risk genes related to KIRC prognosis were screened out, and these genes were mainly involved in immune-related functions such as lymphocyte migration. At the same time, we combined TCGA and GEO to find four genes (BASP1, CCL8, FCGR1B, FKBP11) for determining the risk stratification of KIRC, and constructed a model for clinicians to assess KIRC prognosis based on gene expression and clinical information. qPCR confirmed that BASP1, FCGR1B, and FKBP11 were significantly upregulated in KIRC compared to adjacent normal tissues, whereas CCL8 showed no significant differential expression between KIRC and paracancerous tissues.

**Conclusion:**

Our study has the potential to assist clinicians assess KIRC prognosis and modify more appropriate personalized treatment for KIRC patients in a timely manner.

## Introduction

Globally, over 400,000 people are affected by renal cell carcinoma (RCC) each year (Bray et al., 2018). RCC has various subtypes, but kidney renal clear cell carcinoma (KIRC) is the most common, accounting for approximately 70% of all RCC cases (Jonasch et al., 2021). Although many patients with KIRC can be treated with radical surgery at an early stage, there are also many patients who are already locally advanced or metastasized at the time of diagnosis of KIRC, and these patients do not respond well (Cotta et al., 2023). In addition, even KIRC patients at the same stage and grade with the same treatment may have a completely different prognosis (Wei et al., 2019; Zou and Mo, 2021), suggesting KIRC is a heterogeneous cancer. Given the prognostic differences among KIRC patients, proper risk stratification is essential for effectively identifying which patients require more intensive initial treatment, closer follow-up, and timely adjustments to more effective treatment regimens. Therefore, it is important to find a risk stratification method with high predictive value to enhance the outcome of KIRC. With the advancement of various molecular profiling technologies, such as transcriptome sequencing and novel bioinformatics analysis tools, researchers are now able to study tumor biology in greater depth and stratify patients based on characteristics associated with clinical outcomes.

Changes in the tumor immune microenvironment (TIM) significantly impact tumor development, progression, and prognosis (Fridman et al., 2017; Liu et al., 2025). Previous studies have shown that the proportion of immune cells and immune-related genes in cancer tissues leads to the difference in prognosis of cancer patients (Zeng et al., 2019; Zhang et al., 2019). Although there has been an increase in the number of studies exploring KIRC prognosis from an immunological perspective in recent years, there are still few studies that integrate multiple databases to stratify the immune-related risk of KIRC and accurately predict the prognosis of KIRC.

In this study, we first integrated the transcriptomic data of KIRC from GSE404435 and GSE46699 for immune classification and compared the differences in gene expression and tumor microenvironment between the two immune subtypes. We then combined these findings with TCGA’s KIRC data for risk stratification and verified the expression of the genes used for risk stratification in KIRC and adjacent normal tissues by quantitative real-time PCR (qPCR). Finally, we constructed a model incorporating risk scores and clinical information to aid clinicians in predicting the prognosis of KIRC patients.

## Methods

### Collection and collation of GSE40435 and GSE46699 data from GEO database

We first obtained the gene expression matrix of GSE40435 and GSE46699 through the “Biobase” and “GEOquery” packages of R language, and removed the normal samples according to the clinical information. Finally, the gene expression matrix of 101 cancer samples from GSE40435 and 67 cancer samples from GSE46699 were obtained. Tumor samples from GSE40435 and GSE46699 data were scored by single-sample gene set enrichment analysis (ssGSEA), and were divided into high immune score group (Immunity_H) and low immune score group (Immunity_L) according to the Euclidean distance and immune score (Guan et al., 2020). ssGSEA is a method to comprehensively score immune characteristics of samples based on 29 immune-related genes or functions (Wu et al., 2022). Then we used the “estimate” package of R software to evaluate the tumor microenvironment of the samples of Immunity_H and Immunity_L groups. Moreover, we compared the expression of human leukocyte antigen (HLA) related genes in Immunity_H and Immunity_L groups. The differentially expressed genes (DEGs) of the Immunity_H and Immunity_L groups were calculated using the Immunity_L as reference. *P* < 0.05 and absolute value of logFC >0.585 were used as the threshold of DEGs.

### Acquisition and processing of KIRC data from TCGA

Clinical and transcriptomic data information for KIRC was downloaded from TCGA, and the genetic data matrix containing only KIRC (excluding normal samples) was obtained through strawberry perl software and “limma” package in R language. Then we obtained the matrix of DEGs upregulated in Immunity_H of GSE40435 and GSE46699 datasets in TCGA. At the same time, by combining the genetic data matrix and survival data of KIRC samples from TCGA, the genes that have obvious influence on KIRC prognosis were obtained. Through analyzing protein-protein interactions (PPI) of prognostic related genes (PRGs) and their co-expression with tumor related transcription factors (TFs), the correlation between these PRGs and the possible mechanisms affecting KIRC prognosis were further explored. A correlation coefficient absolute value greater than 0.4 and an FDR less than 0.001 were used as thresholds for screening co-expression between PRGs and TFs. Absolute value of correlation coefficient (|cor| >0.4) and an FDR (FDR <0.001) were used as thresholds for screening co-expression between PRGs and TFs.

### Integration and processing of TCGA and GEO data

Utilizing survival data and clinical information of 530 KIRC patients from TCGA, along with PRGs obtained from TCGA and GEO datasets, we developed a risk stratification using lasso Cox regression analysis, leveraging the four genes expression profiles to predict patient outcomes in KIRC. Based on these four genes expression levels, KIRC samples were stratified into high-risk and low-risk groups employing the median score, and the survival prognosis of the two groups was compared. The risk score is calculated using the following formula: Risk score = $\Sigma_{k}^{n}$ Expression_(k)_ ✕ coef_(k)_. Expression_(k)_ = expression level of gene “k” in KIRC samples. coef_(k)_ = regression coefficient of feature gene “k”. These four genes and their corresponding coefficients are shown in Supplementary Table S1.

### GeneMANIA, TIMER and UALCAN

The functional networks of PRGs were analyzed using GeneMANIA (Franz et al., 2018). TIMER is a powerful database that can be used to evaluate immune infiltration in different aspects of multiple cancers (Li et al., 2017). We assessed the association between the four prognostic model genes and immune cell infiltration levels in KIRC using TIMER. UALCAN is a multifunctional database that can be used to evaluate the expression of multiple genes in different cancers and their impact on cancer prognosis (Chandrashekar et al., 2022). We used the “scan by genes” feature in the UALCAN database to the expression of the four genes used for risk stratification in KIRC and their impact on the prognosis of KIRC.

### Tumor mutation burden

Tumor mutation burden (TMB), a known predictor of therapeutic response and survival outcomes (Provencio et al., 2023; Sung et al., 2022). We assessed TMB differences between high- and low-risk KIRC groups by “limma” and “ggpubr” packages, and the effect on the prognosis of KIRC was evaluated by TMB and risk stratification.

### Construction of a prognostic model

Compile the clinical information of KIRC patients and exclude those with unclear data. For the remaining 526 KIRC patients (with clearly defined gender, age, stage, grade, and risk stratification), construct a prognostic model and perform an independent prognostic analysis of risk stratification using the “survival”, “survminer”, and “TimeROC” packages.

### qPCR

We collected samples from four KIRC patients at the Affiliated Hospital of Guizhou Medical University and compared the expression differences of four risk-score-related genes between KIRC tissues and adjacent normal tissues using qPCR. Isolate total RNA from tissue using TRIzol. After verifying RNA purity, we conducted reverse transcription to generate cDNA. Following polymerase chain reaction (PCR), the relative expression levels of target genes in tumor tissues compared with adjacent normal tissues were analyzed using the 2^−ΔΔCt^ method, with each sample assayed in triplicate. qPCR was conducted using the primer sequences listed below.

GAPDH:

5′-AATCAAGTGGGGCGATGCTG-3' (Forward), 5′-GCAAATGAGCCCCAGCCTTC-3′ (Reverse);

BASP1 (Brain Abundant Membrane Attached Signal Protein 1):

5′-AGGGGAACCCAAAAAGACTGA-3' (Forward),

5′-GGTGTGGAACTAGGCGCTTC-3' (Reverse); CCL8 (C-C Motif Chemokine Ligand 8):

5′-TGGAGAGCTACACAAGAATCACC-3' (Forward),

5′-TGGTCCAGATGCTTCATGGAA-3' (Reverse); FCGR1B (Fc Gamma Receptor Ib):

5′-AGTTGATGGGCAAGTGGACAC-3' (Forward), 5′-TCTCTGGCACCTGTATTCACC-3' (Reverse); FKBP11 (FKBP Prolyl Isomerase 11):

5′-GAGAAGCGAAGGGCAATCATT-3' (Forward),

5′-GATGGTGGAAATCCCCGTTTT-3' (Reverse).

### Statistical analysis

All statistical analyses were performed using R software (version 4.5.1), with categorical variables compared between groups using the chi-square test (chisq.test), while continuous variables were analyzed using non-parametric tests, including the Mann-Whitney-Wilcoxon test (wilcox.test) for two-group comparisons and the Kruskal–Wallis H test (kruskal.test) for multi-group comparisons; a p-value <0.05 was considered statistically significant.

## Results

### Immunotyping of tumor samples from GSE40435 and GSE46699

**FIGURE 1:**
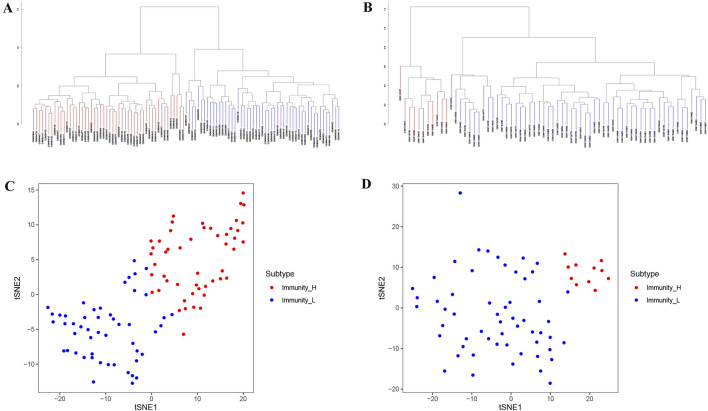
Immune clustering and stratification of KIRC patients from GEO data. **(A,B)** The systematic clustering and immune grouping plots of KIRC patients from GSE40435 **(A)** and GSE46699 **(B)** based on ssGSEA analysis. **(C,D)** The results of tSNE from GSE40435 **(C)** and GSE46699 **(D)** further confirmed the reliability of systematic clustering of KIRC patients.

We performed ssGSEA scoring on tumor samples from GSE40435 and GSE46699, respectively, and classifying them into two immune-related subtypes: Immunity_H and Immunity_L based on the scores, hierarchical clustering and tSNE algorithm. Hierarchical clustering and tSNE showed similar results, that was, Immunity_L and Immunity_H could be well distinguished (Figures 1A–D). The GSE40435 cohort (n = 101) was stratified into 51 Immunity_L and 50 Immunity_H samples, while GSE46699 (n = 67) contained 57 Immunity_L and 10 Immunity_H samples (Supplementary Material S1,S2).

### Comparison of the characteristics of two immunophenotypes

**FIGURE 2:**
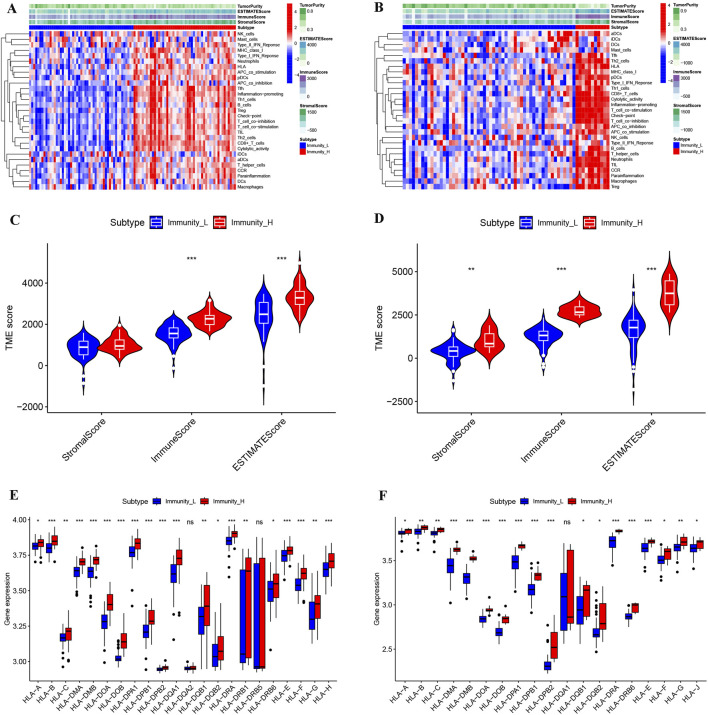
Comparison of the microenvironment across different immune subtypes of KIRC. **(A,B)** Landscape of microenvironments of different immune subtypes from the GSE40435 **(A)** and GSE46699 **(B)** cohorts. **(C,D)** StromalScore, ImmuneScore and ESTIMATEScore of different subtypes from the GSE40435 **(C)** and GSE46699 **(D)** cohorts. **(E,F)** HLA gene expression levels from different subtypes of GSE40435 **(E)** and GSE46699 **(F)** cohorts.

**FIGURE 3:**
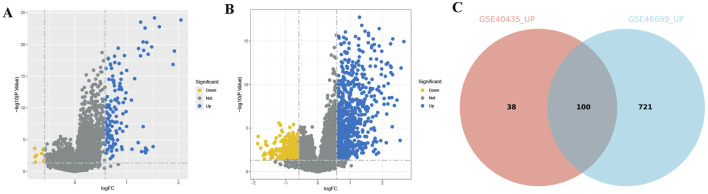
DEGs of different immune subtypes in KIRC. **(A,B)** Volcano maps of DEGs from different subtypes of the GSE40435 **(A)** and GSE46699 **(B)** cohorts. **(C)** Venn diagram of DEGs upregulated from both GSE40435 and GSE46699 cohorts (Immunity_L vs. Immunity_H, Immunity_L as the control group).

After immunotyping the KIRC samples, we compared the immune-related characteristics of Immunity_H and Immunity_L groups in GSE40435 and GSE46699, respectively. In the GSE40435 and GSE46699 data, the Immunity_H group demonstrated significantly higher scores for immune-related functions compared to the Immunity_L group (Figures 2A,B). Furthermore, both the ImmuneScore and the ESTIMATEScore of the Immunity_H were significantly higher than those of the Immunity_L (Figures 2C,D). Additionally, HLA-related genes showed significantly elevated expression in Immunity_H compared to Immunity_L (Figures 2E,F). These findings demonstrate that KIRC immunotyping (Immunity_L vs. Immunity_H) captures significant heterogeneity in tumor immune microenvironments. Comparative transcriptomic analysis revealed significant upregulation of multiple differentially expressed genes (DEGs) in Immunity_L vs. Immunity_H across both GSE40435 and GSE46699 datasets (Figures 3A,B). By taking the intersection of DEGs in GSE40435 and GSE46699 data, we found 100 co-upregulated DEGs in the Immunity_H group from the two datasets (Figure 3C).

### PRGs and their regulatory networks

**FIGURE 4:**
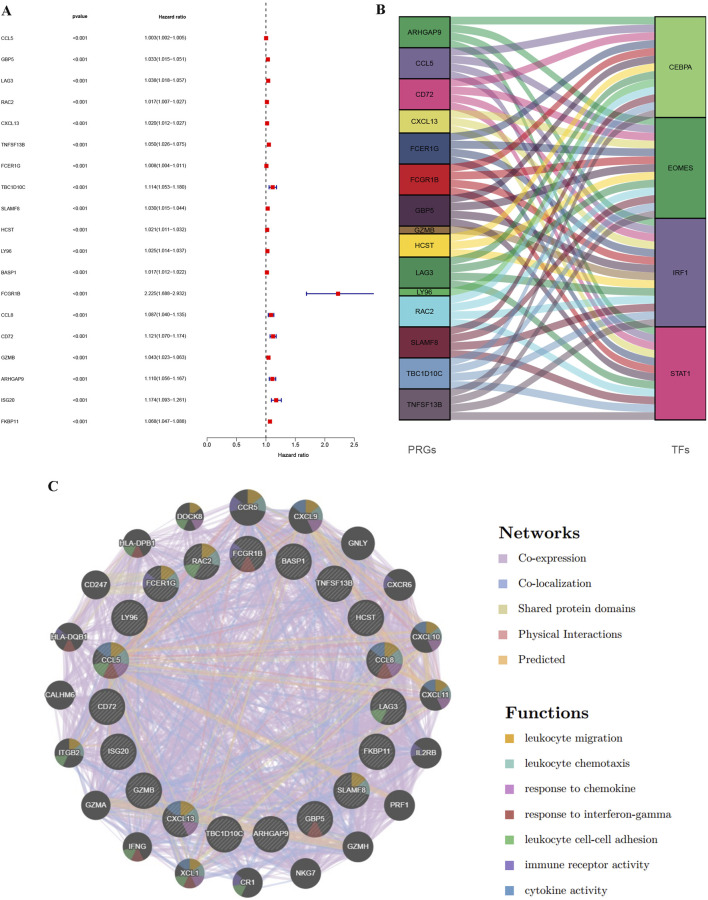
Identification of PRGs in KIRC through integration of TCGA and GEO data and regulatory network construction. **(A)** Forest map of genes significantly upregulated in the GEO database (GSE40435 and GSE46699) and significantly affecting survival time in TCGA-KIRC patients. **(B)** Alluvial map of PRGs and TFs. **(C)** PPI interaction network diagram between PRGs.

The analysis results obtained by integrating multiple datasets are more reliable, so we further incorporated the TCGA database to explore KIRC. We integrated expression profiles of 100 consistently upregulated genes (identified in both GSE40435 and GSE46699) with TCGA-KIRC clinical data to identify PRGs signatures. Survival analysis identified 19 high-risk PRGs (Hazard ratio, HR >1, *p* < 0.001) significantly associated with poorer outcomes in KIRC patients (Figure 4A). Co-expression analysis revealed unexpected universal positive correlations between all screened PRGs and differentially expressed TFs in Immunity_L vs. Immunity_H comparisons (Figure 4B, Supplementary Material S3). To further explore the possible mechanisms by which PRGs affect KIRC, we explored the protein-protein interaction network of PRGs. The main functions of these PRGs included leukocyte migration, leukocyte chemotaxis, response to chemokine, response to interferon-gamma, leukocyte cell-cell adhesion, immune receptor activity and cytokine activity (Figure 4C). These results suggest that the difference of immune microenvironment is an important reason for the difference in prognosis of patients with KIRC.

### Risk stratification for KIRC patients by combining data from GEO and TCGA

**FIGURE 5:**
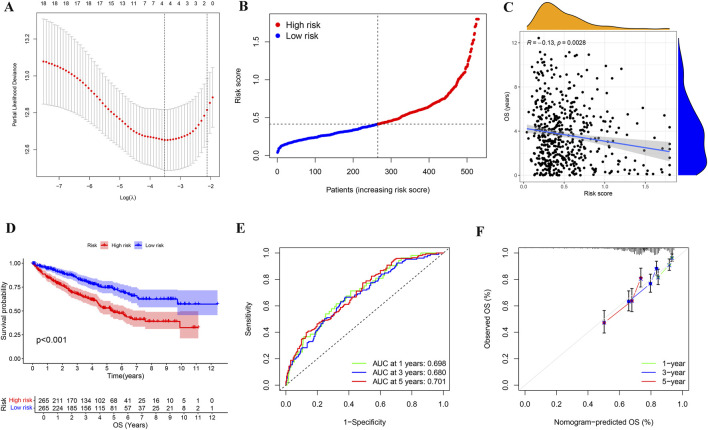
Identification and analysis of risk stratification in KIRC patients. **(A)** Cross-validation map of gene parameter selection in a lasso model. **(B)** Risk score distribution map for KIRC patients based on the selected four genes. **(C)** Correlation analysis of risk score and OS in KIRC patients. **(D)** Kaplan-Meier survival analysis of KIRC in different risk groups. **(E)** The AUC for 1-year, 3-year, and 5-year survival predictions of ROC analysis based on the lasso model were 0.698, 0.68, and 0.781, respectively. **(F)** Calibration plot for constructing KIRC risk signature based on genes selected by the lasso model. OS: overall survival.

To further investigate KIRC, we reduced the dimension of PRGs by lasso regression analysis (Figure 5A), obtained four genes for assessing KIRC risk, and divided KIRC into high and low risk groups based on the gene expression and the median risk score (Figure 5B). Subsequent investigation showed a negative correlation between the risk score and the KIRC patients’ overall survival (Figure 5C). Moreover, the survival analysis further confirmed a significant difference in survival time between the high-risk and low-risk groups, with the high-risk group having a shorter survival time (Figure 5D). We performed ROC analysis based on the predicted survival time of patients to better determine the superiority of risk stratification in predicting survival. ROC analysis demonstrated AUCs of 0.698 (1-year), 0.680 (3-year), and 0.781 (5-year) for survival prediction (Figure 5E). Calibration plots confirmed strong agreement between predicted and observed outcomes across all timepoints (Figure 5F).

**FIGURE 6:**
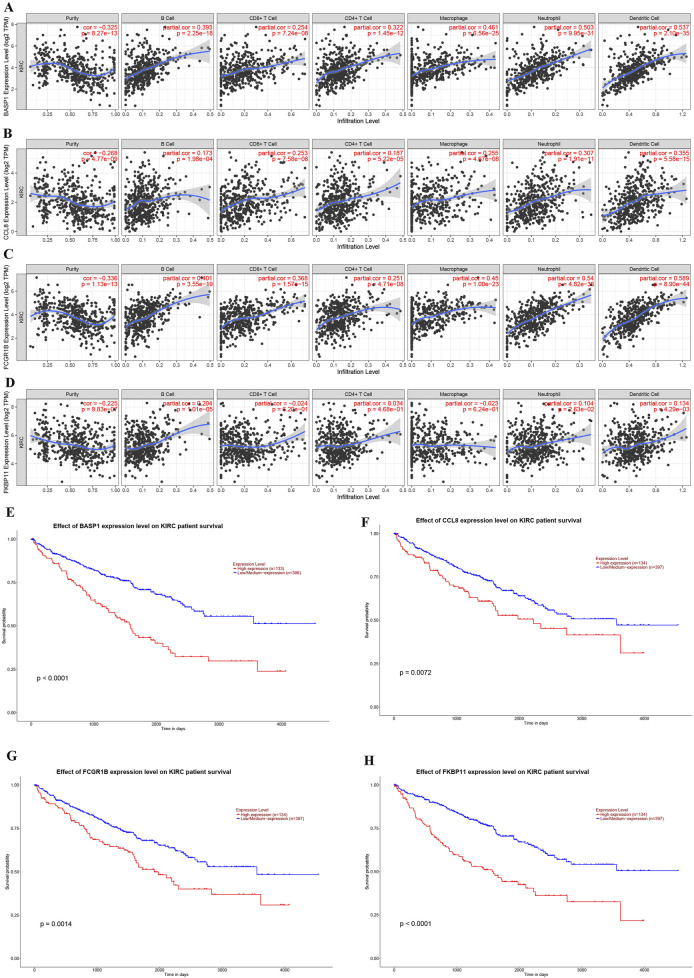
Correlations of gene expression levels for KIRC risk stratification with immune cell infiltration and their effects on KIRC prognosis. **(A–D)** Association between the expression levels of BASP1 **(A)**, CCL8 **(B)**, FCGR1B **(C)** and FKBP11 **(D)** genes and immune cell infiltration in KIRC. **(E–H)** Effects of BASP1 **(E)**, CCL8 **(F)**, FCGR1B **(G)** and FKBP11 **(H)** gene expression levels on the prognosis of KIRC.

Immune infiltration analysis revealed significant associations between the four genes (BASP1, CCL8, FCGR1B, FKBP11) used for risk stratification and tumor microenvironment composition in KIRC (Figures 6A–D). In addition, we also verified that the high expressions of BASP1, CCL8, FCGR1B and FKBP11 in KIRC were not conducive to the survival and prognosis of patients through UALCAN database (Figures 6E–H). These results suggest that the difference of immune microenvironment is an important factor affecting the prognosis of KIRC patients.

### The expression of risk stratification genes in KIRC and adjacent normal tissues

**FIGURE 7:**
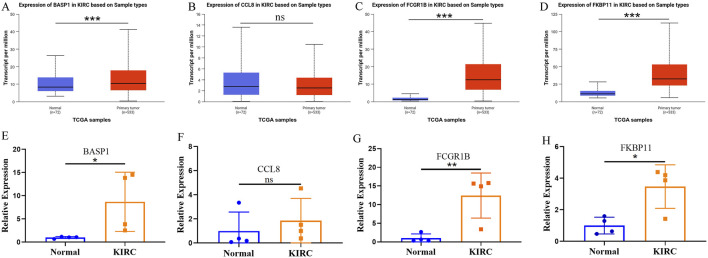
The expression of risk stratification genes in KIRC and adjacent normal tissues. **(A–D)** The UALCAN database shows the expression of BASP **(A)**, CCL8 **(B)**, FCGR1B **(C)** and FKBP11 **(D)** in KIRC and adjacent normal tissues. **(E–H)** The expressions of BASP **(E)**, CCL8 **(F)**, FCGR1B **(G)** and FKBP11 **(H)** in KIRC and adjacent normal tissues were verified by qPCR.

Subsequently, we utilized the UALCAN database to investigate the expression patterns of risk-stratification genes in KIRC and adjacent normal tissues. The analysis revealed significantly higher expression levels of BASP1, FCGR1B, and FKBP11 in KIRC compared to normal adjacent tissues, while CCL8 showed no significant differential expression between tumor and non-tumor tissues (Figures 7A–D). Furthermore, our qPCR experimental results validated these expression patterns for all four genes (Figures 7E–H). These results underscore the importance of comprehensive multi-gene evaluation for risk stratification in KIRC.

### Tumor mutation burden and risk stratification combined to determine the KIRC prognosis

**FIGURE 8:**
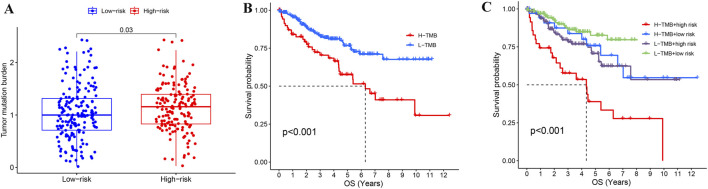
Combined analysis of TMB and risk stratification in KIRC. **(A)** Comparison of TMB between high-risk and low-risk groups in KIRC. **(B)** Effect of TMB on the prognosis of KIRC. **(C)** Combining TMB and risk stratification to assess KIRC prognosis.

After clarifying the significance of risk stratification for KIRC patients, we also evaluated the tumor mutation burden (TMB) in high and low risk groups. The TMB in the high-risk group was prominently higher than in the low-risk group (Figure 8A), and KIRC patients with a high TMB (H-TMB) had a worse survival prognosis than those with low TMB (L-TMB) (Figure 8B). We also assessed the prognosis of KIRC patients in combination with TMB and risk stratification, and found that KIRC patients in the L-TMB and low-risk group had the best survival outcomes, while those in the H-TMB and high-risk group had the worst survival outcomes (Figure 8C).

### Independent prognostic assessment and construction of prognosis model of KIRC patients based on risk score and clinical information

**FIGURE 9:**
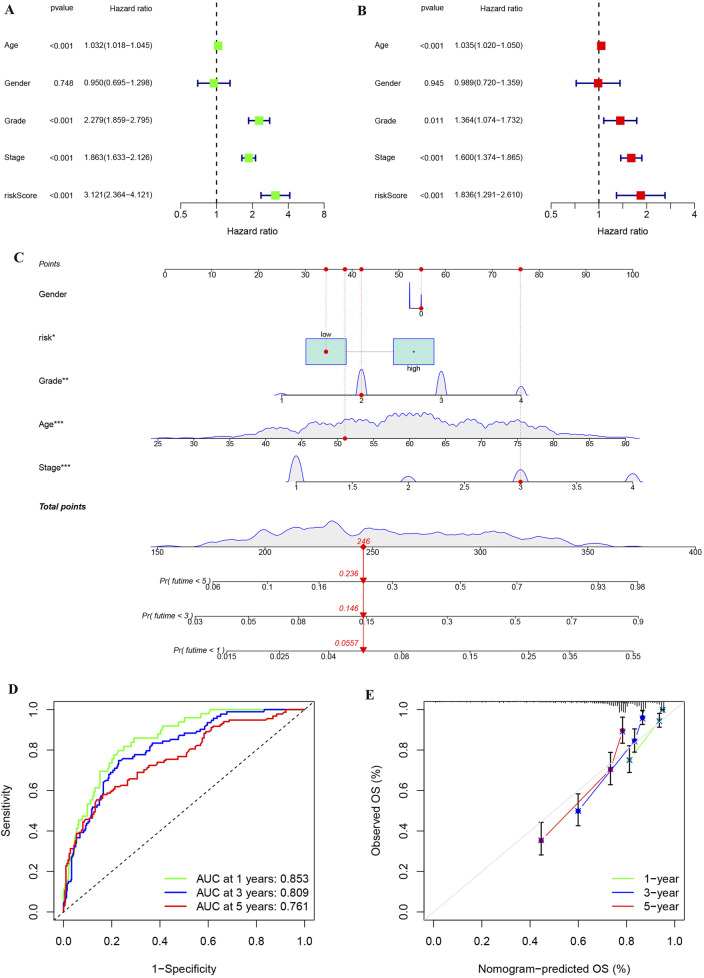
Univariate and multivariate analyses on risk stratification and constructing and assessing a nomogram model of KIRC. **(A)** Univariate cox regression analysis of risk stratification and other variables in KIRC. **(B)** Multivariate cox regression analysis of risk stratification and other variables in KIRC. **(C)** Construction of a nomogram based on risk stratification and clinical features to aid clinicians in determining KIRC prognosis. **(D)** ROC analysis for nomogram accuracy. **(E)** Calibration diagram for evaluating similarity of nomogram to ideal model.

Univariate and multivariate Cox regression analyses were performed to assess whether the risk score provides independent prognostic value beyond conventional clinical parameters (stage, grade, age, and gender) in KIRC patients. Both univariate and multivariate analyses showed that risk score could be used as an independent prognostic factor in KIRC patients, and its HR value was higher than that of stage, grade and age (Figures 9A,B). To facilitate clinical implementation, we developed a prognostic nomogram integrating standard clinical parameters (age, gender, TNM stage, grade) with our molecular risk score for individualized KIRC outcome prediction. By combining various clinical characteristics of KIRC patients into a comprehensive score, linear trend analysis revealed a significant positive association between composite risk scores and mortality risk, with higher scores predicting poorer clinical outcomes (Figure 9C). ROC analysis confirmed the nomogram’s strong predictive accuracy for patient survival (Figure 9D), while calibration plots demonstrated excellent agreement between predicted and observed outcomes (Figure 9E).

## Discussion

KIRC is a disease that can be effectively treated through surgery in its early stages (Jonasch et al., 2021). However, up to one-third of patients progress to advanced KIRC (Jonasch et al., 2014), and the prognosis varies significantly among patients (Wu et al., 2022). Therefore, further exploration of KIRC subtypes and progression is essential for improved follow-up and treatment. This study undertook a comprehensive analysis of KIRC gene expression data and tumor immune-related features from the GEO and TCGA datasets. We investigated and compared the immune profiling of KIRC, performed risk stratification, and developed a prognostic model. This model is designed to assist clinicians in predicting the prognosis of KIRC patients, facilitating more effective follow-up and the creation of personalized treatment plans.

KIRC is a highly immunogenic tumor mediated by immune cells and their related secretory factors (Chen et al., 2023). Immune checkpoint inhibitors benefit only a subset of KIRC patients (Diaz-Montero et al., 2020), likely due to the heterogeneity of the TIM (Vesely et al., 2022). TIM can influence tumor progression and prognosis by releasing various cytokines and products (Varricchi et al., 2017; Liu and Yang, 2021). Given this biological complexity and the variable therapeutic responses observed clinically, there is an urgent need for robust, TIM-derived biomarkers to improve risk stratification. In our study, according to ssGSEA analysis, KIRC samples from the two cohorts were stratified into Immunity_H and Immunity_L groups. We found significant differences in the expression of multiple immune-related genes and the composition of the immune microenvironment between the two immune subtypes. These results indicate that it is necessary and reasonable to divide KIRC into these two immune subtypes. We performed comparative analysis of differentially expressed genes (DEGs) between the two immune subtypes and conducted integrative analysis of GEO and TCGA cohorts to identify multiple PRGs significantly associated with KIRC outcomes. The co-expression of PRGs and TFs, along with gene enrichment analysis, suggests that these PRGs are mainly involved in lymphocyte migration, lymphocyte chemotaxis, response to interferon-gamma and other immune-related functions. Studies have shown that the migration of lymphocytes is related to the metastasis and progression of tumors (Shi et al., 2021). Lymphocyte chemotaxis can influence the tumor microenvironment through paracrine and autocrine mechanisms, directly affecting tumor cells and playing a crucial role in tumor progression and invasion (Hart et al., 2020). Response to interferon-gamma can serve as effective prognostic indicators for KIRC (Liu et al., 2021). These results indicate that the PRGs we identified are highly reliable and can influence the prognosis of KIRC through multiple immune-related functions. Previous studies primarily screened PRGs using a single database (Wang et al., 2022; Fu et al., 2022). In contrast, we integrated different databases and combined various GEO cohorts, which enhances the superiority of our identified PRGs.

We further classified KIRC patients into high-risk and low-risk groups based on the expression of some PRGs to determine the prognostic risk of patients independent of clinical and pathological data. Patients in the high-risk group had significantly worse outcomes than those in the low-risk group. Notably, KIRC patients with identical clinicopathological staging frequently exhibit marked heterogeneity in clinical outcomes (Wei et al., 2019; Zou and Mo, 2021; Gui et al., 2023; Rini et al., 2015), so it is meaningful for us to further stratify the risk based on patient gene expression.

TIM is a key component in the efficacy of immunotherapy for cancer patients (Braun et al., 2021). Understanding the gene composition of the microenvironment in KIRC patients is essential for precise treatment. The four genes (BASP1, CCL8, FCGR1B, FKBP11) showed consistent positive correlations with immune cell infiltration (B cells, macrophages, dendritic cells, CD8^+^/CD4^+^ T cells, neutrophils) in KIRC. Notably, elevated CD8^+^ T cell abundance was strongly associated with reduced disease-free survival and overall survival (OS) (Dai et al., 2021). It was reported that macrophage infiltration of KIRC was significantly increased in high-risk group (Jiang et al., 2024). Elevated tumor-infiltrating CD4^+^ T cell levels correlated with reduced OS and progression-free survival in KIRC patients. (Correction et al., 2020). Dendritic cell infiltration in KIRC is a double-edged sword, and the infiltration of dendritic cells in the proximal and distal tumor has diametrically opposite prognosis for PFS and OS in KIRC (Xu et al., 2023). Increased B-cell infiltration of CD20^+^ is closely associated with poor prognosis of KIRC (Sjoberg et al., 2018). KIRC patients with increased neutrophil infiltration were less sensitive to immunotherapy (Ding et al., 2023). High expression of four genes used to construct prognostic models is associated with poor prognosis for KIRC. Taken together, our results are similar to previous reports, adding to the reliability of our results.

Moreover, we developed an integrated prognostic score combining clinical parameters and molecular signatures, enabling more accurate and efficient KIRC outcome prediction in clinical practice. Nomogram can provide personalized prognostic information for patients (Wu et al., 2020; Zhang et al., 2023), and our nomogram offers superior performance and a broader range of predictive scores.

Our study has certain limitations, the number of samples used for qPCR verification is not enough. Furthermore, the predictive performance of the nomogram requires additional validation in prospective cohorts. Meanwhile, the biological functions of the identified target genes should be further investigated through complementary *in vivo* and *in vitro* experiments. Moving forward, our prediction model will need continuous refinement and optimization to enhance its clinical applicability. However, our study highlights the important influence of tumor immune microenvironment on the prognosis of KIRC, explores the key genes that have an important effect on KIRC prognosis, and stratifies KIRC risk according to the key PRGs.

## Conclusion

Using multi-cohort data (GEO and TCGA), we developed a KIRC risk stratification model and implemented it as a clinical nomogram integrating molecular risk scores with key clinicopathological parameters to guide prognosis assessment and therapeutic management. The immune microenvironment is an important factor affecting the prognosis of KIRC.

## Data Availability

The datasets presented in this study can be found in online repositories. The names of the repository/repositories and accession number(s) can be found in the article/Supplementary Material.
