# Supplementary material for: Identification of kidney renal clear cell carcinoma prognosis based on gene expression and clinical information
Source: Front Mol Biosci. 2025 Aug 20;12:1630250. doi: 10.3389/fmolb.2025.1630250 (PMC12405253; doi:10.3389/fmolb.2025.1630250)
Supplement: Supplementary file 1 [file DataSheet1.zip › all raw data/Ethics approval.pdf]

伦理证明

贵州医科大学附属医院泌尿外科邹雄撰写的文章《**Identification of kidney renal clear cell carcinoma prognosis based on gene expression and clinical information**》经医学伦理委员会审核，认为改论文内容不涉及患者隐私及商业利益，符合伦理相关法律法规要求，同意投稿。

贵州医科大学附属医院医学伦理委员会

2025.04.21
