# Supplementary material for: Identification of kidney renal clear cell carcinoma prognosis based on gene expression and clinical information
Source: Front Mol Biosci. 2025 Aug 20;12:1630250. doi: 10.3389/fmolb.2025.1630250 (PMC12405253; doi:10.3389/fmolb.2025.1630250)
Supplement: Supplementary file 1 [file DataSheet1.zip › all raw data/Figures/Figure 4/Figure 4C.pdf]

GeneMANIA report

Created on : 26 May 2024 10:41:30
Last database update : 13 August 2021 00:00:00
Application version : 3.6.0

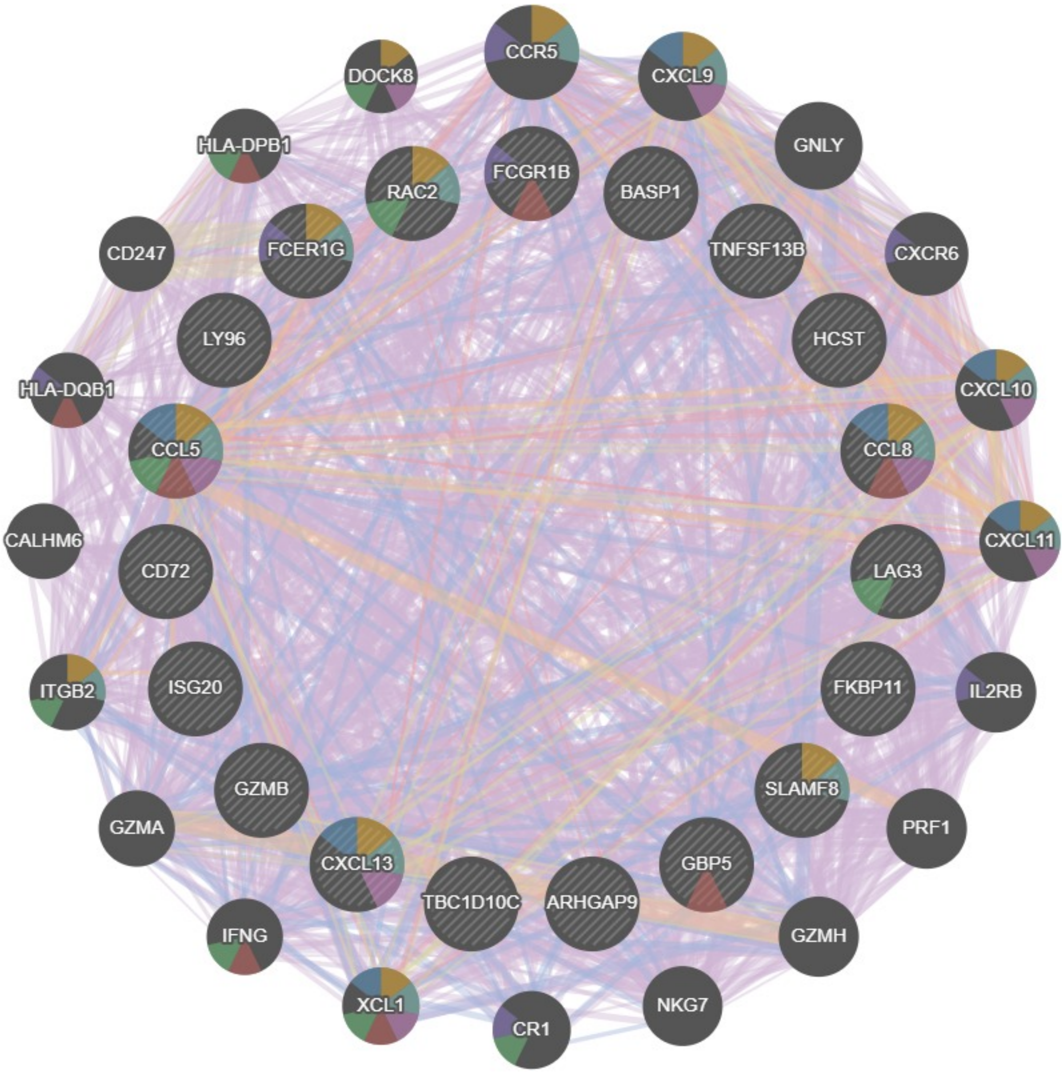

Networks

- Co-expression
- Co-localization
- Shared protein domains
- Physical Interactions
- Predicted

Functions

- leukocyte migration
- leukocyte chemotaxis
- response to chemokine
- response to interferon-gamma
- leukocyte cell-cell adhesion
- immune receptor activity
- cytokine activity
