# Supplementary material for: Identification of kidney renal clear cell carcinoma prognosis based on gene expression and clinical information
Source: Front Mol Biosci. 2025 Aug 20;12:1630250. doi: 10.3389/fmolb.2025.1630250 (PMC12405253; doi:10.3389/fmolb.2025.1630250)
Supplement: Supplementary file 1 [file DataSheet1.zip › all raw data/Table S1.docx]

**Table S1：**

The genes and coefficients used to calculate the risk score for each sample

| Genes | Coefficients |
| --- | --- |
| BASP1 | 0.0102740537160761 |
| FCGR1B | 0.383093585033852 |
| CCL8 | 0.0229644247621379 |
| FKBP11 | 0.0316646386359237 |
