# Supplementary figures and images for: Identification of kidney renal clear cell carcinoma prognosis based on gene expression and clinical information
Source: Front Mol Biosci. 2025 Aug 20;12:1630250. doi: 10.3389/fmolb.2025.1630250 (PMC12405253; doi:10.3389/fmolb.2025.1630250)

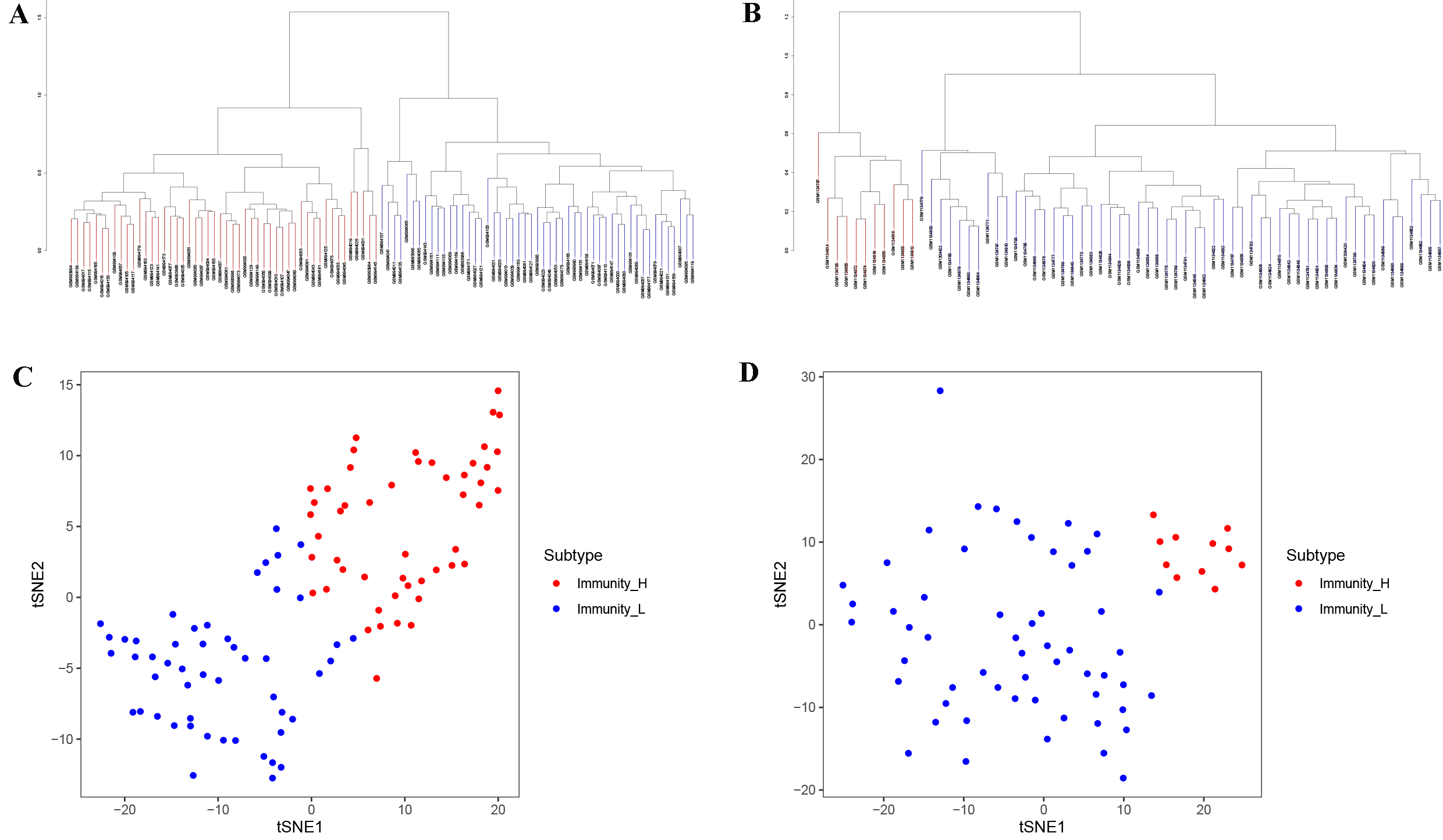

Supplement: Supplementary file 1 [file DataSheet1.zip › all raw data/Figures/Figure/Figure 1.jpg]

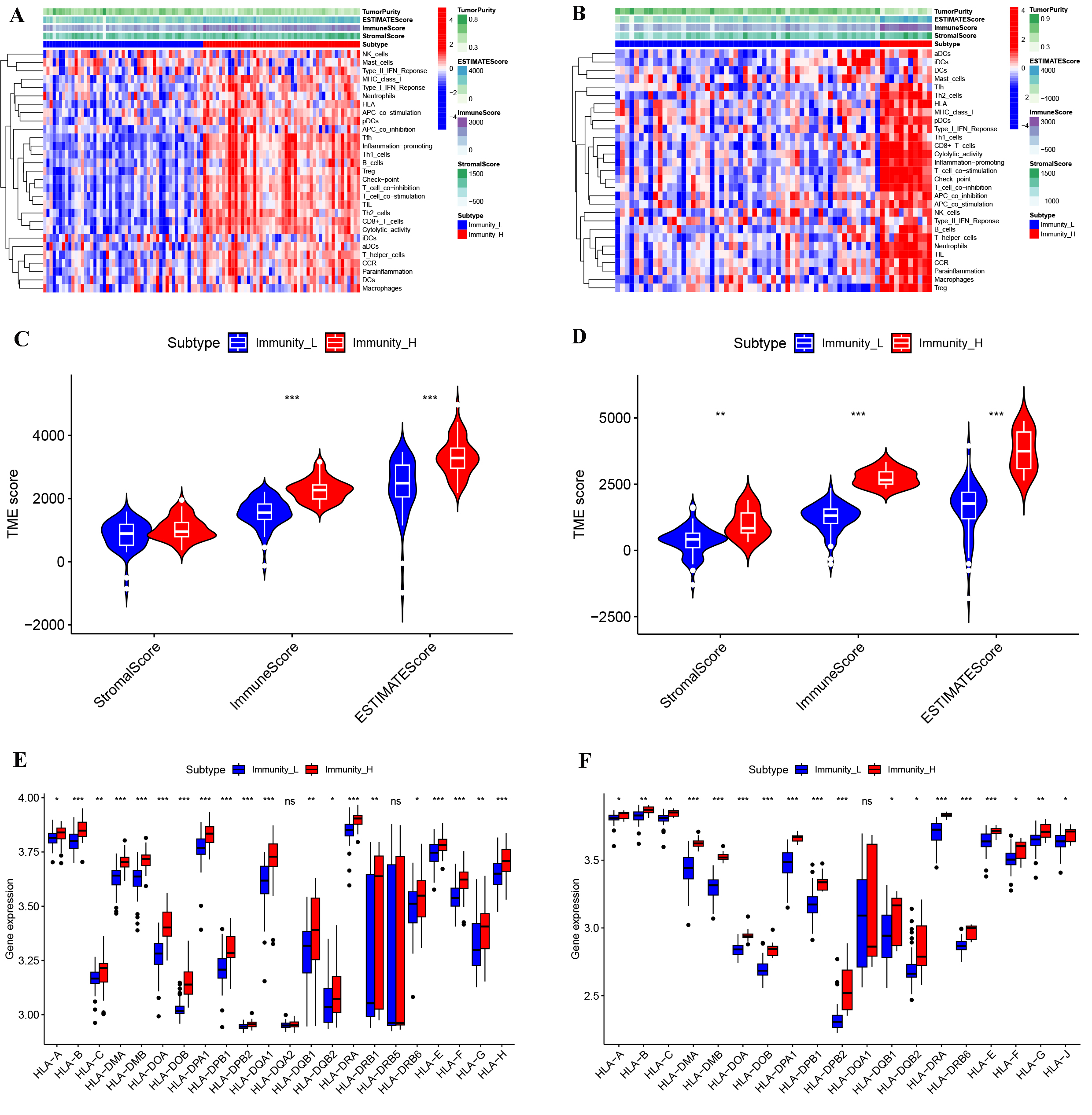

Supplement: Supplementary file 1 [file DataSheet1.zip › all raw data/Figures/Figure/Figure 2.jpg]

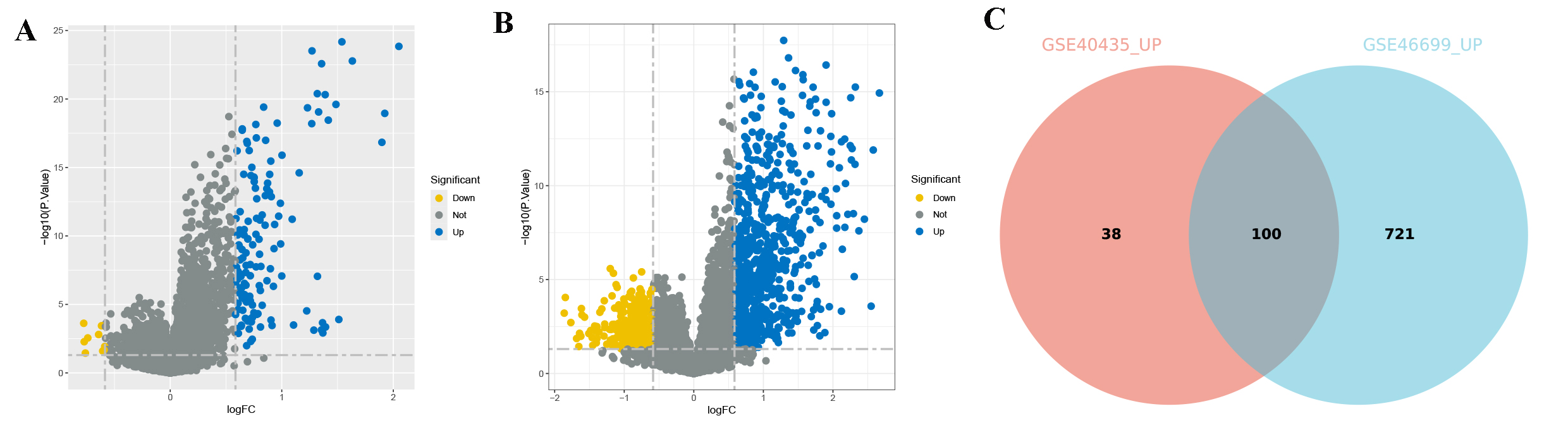

Supplement: Supplementary file 1 [file DataSheet1.zip › all raw data/Figures/Figure/Figure 3.jpg]

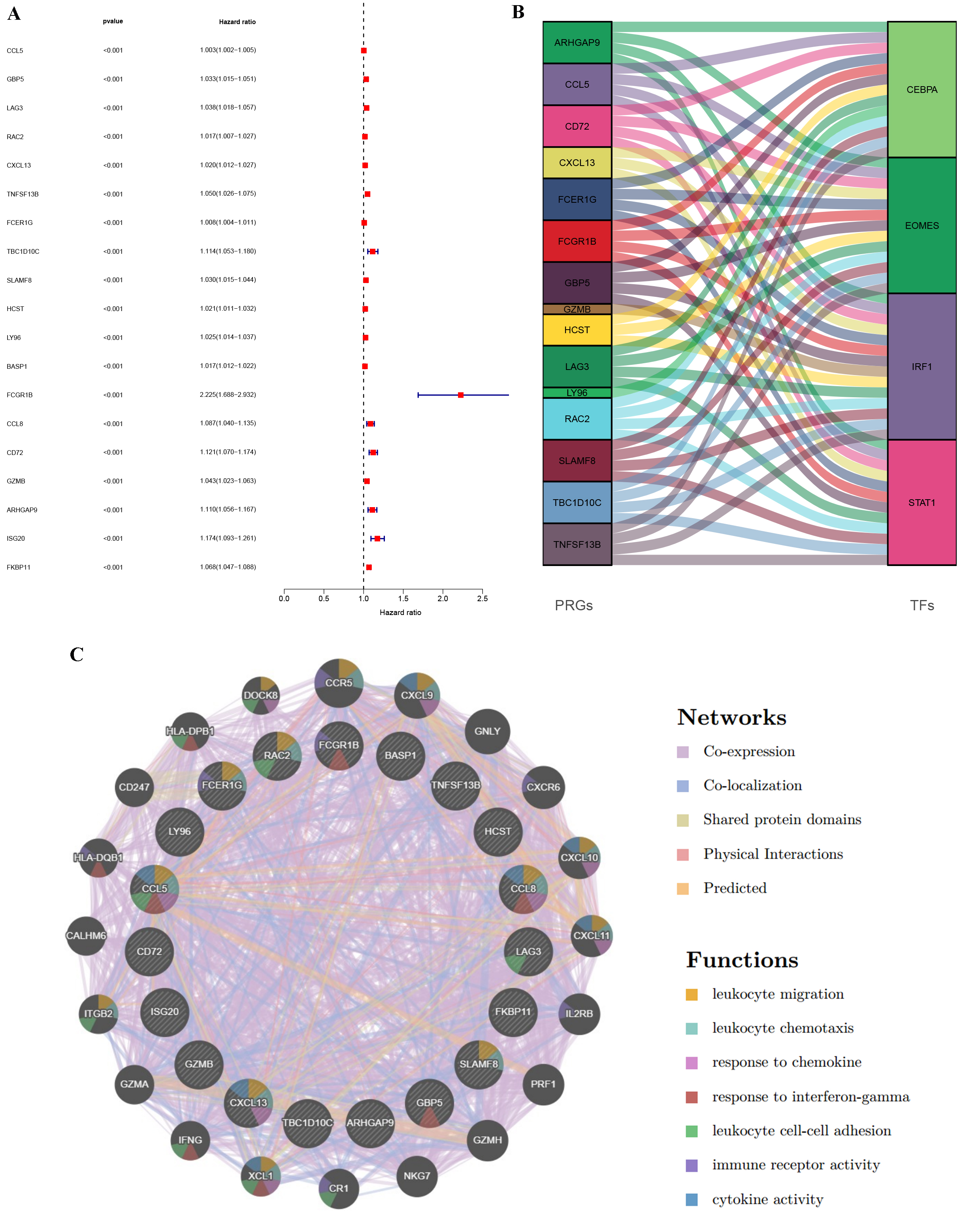

Supplement: Supplementary file 1 [file DataSheet1.zip › all raw data/Figures/Figure/Figure 4.jpg]

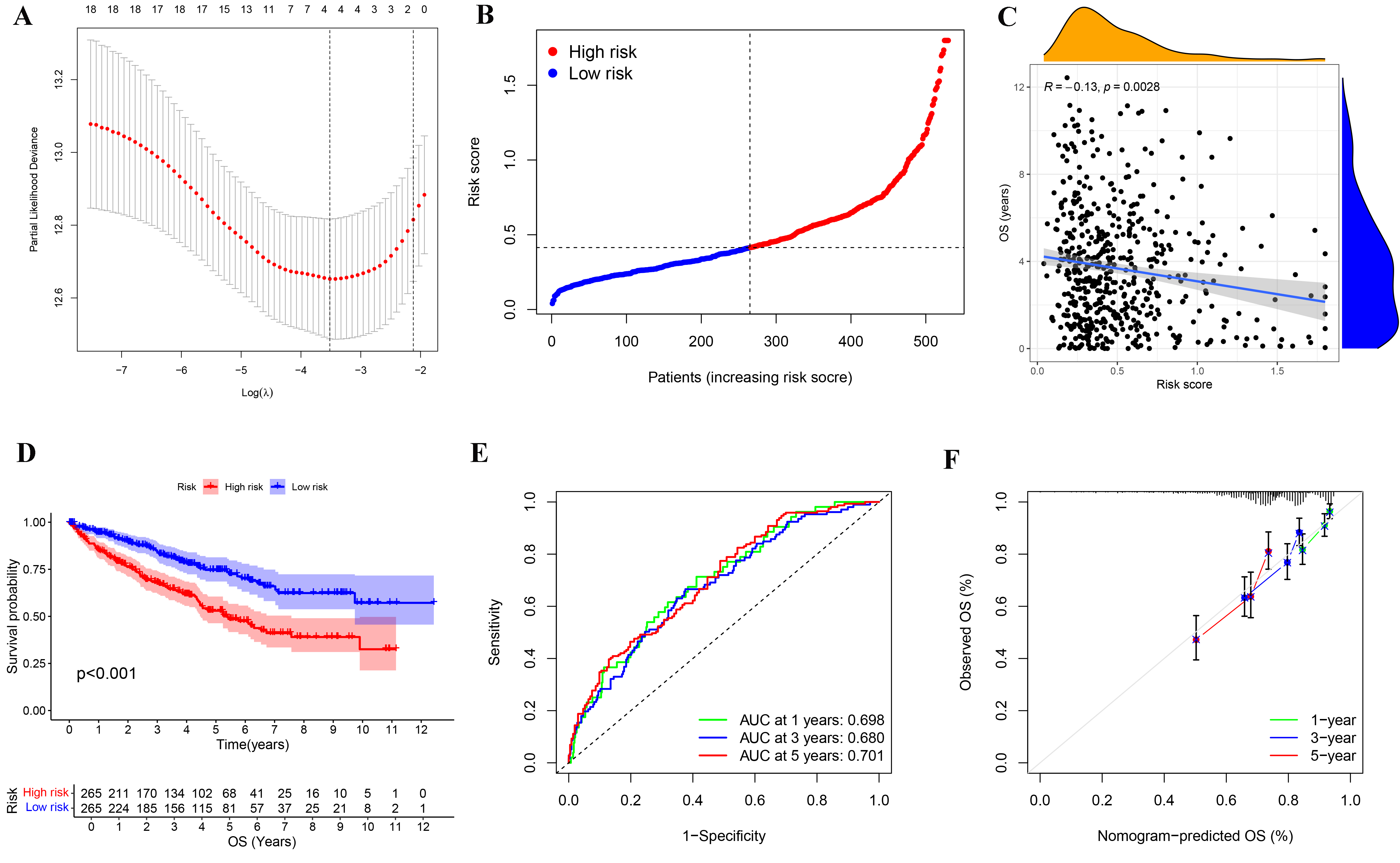

Supplement: Supplementary file 1 [file DataSheet1.zip › all raw data/Figures/Figure/Figure 5.jpg]

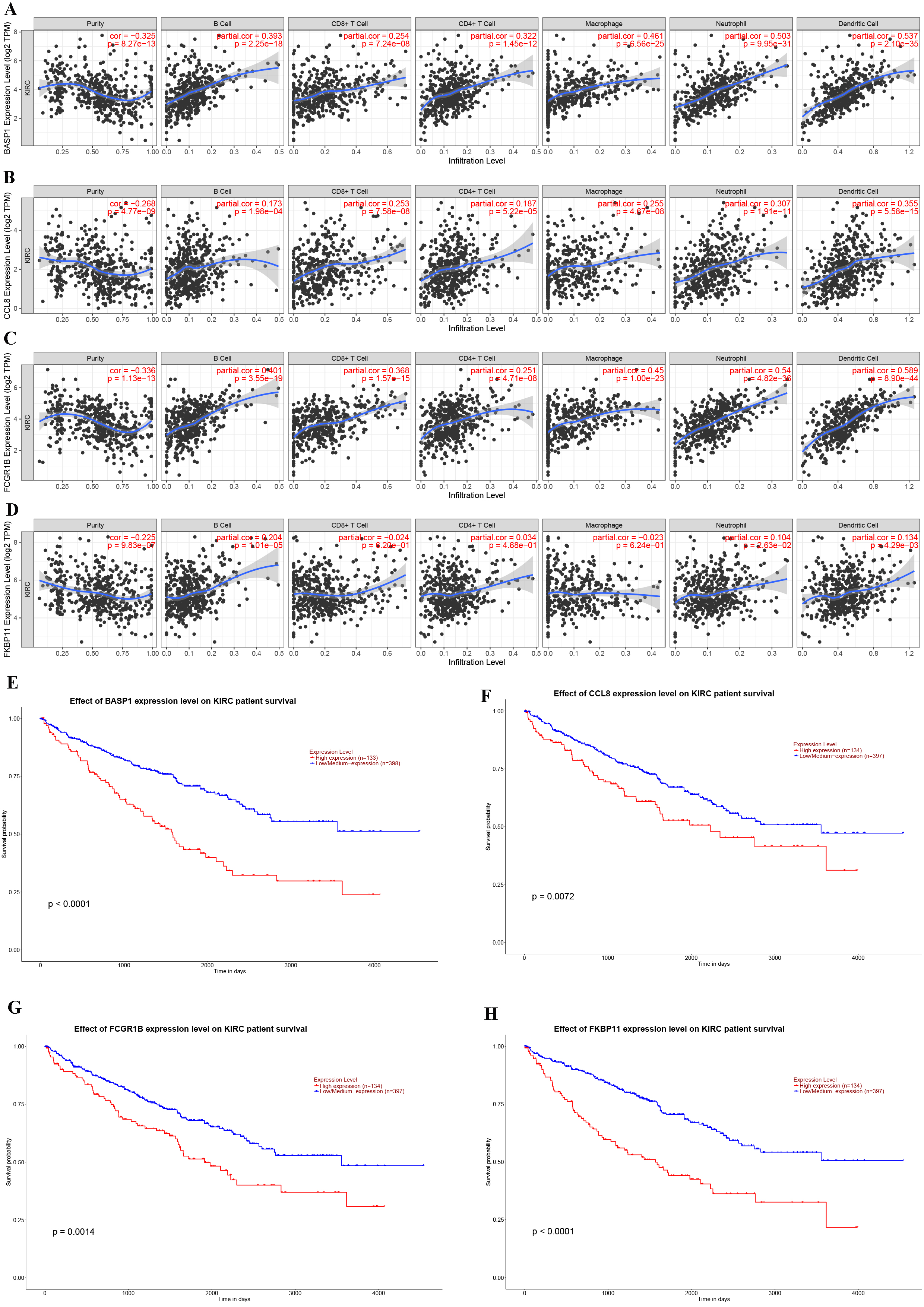

Supplement: Supplementary file 1 [file DataSheet1.zip › all raw data/Figures/Figure/Figure 6.jpg]

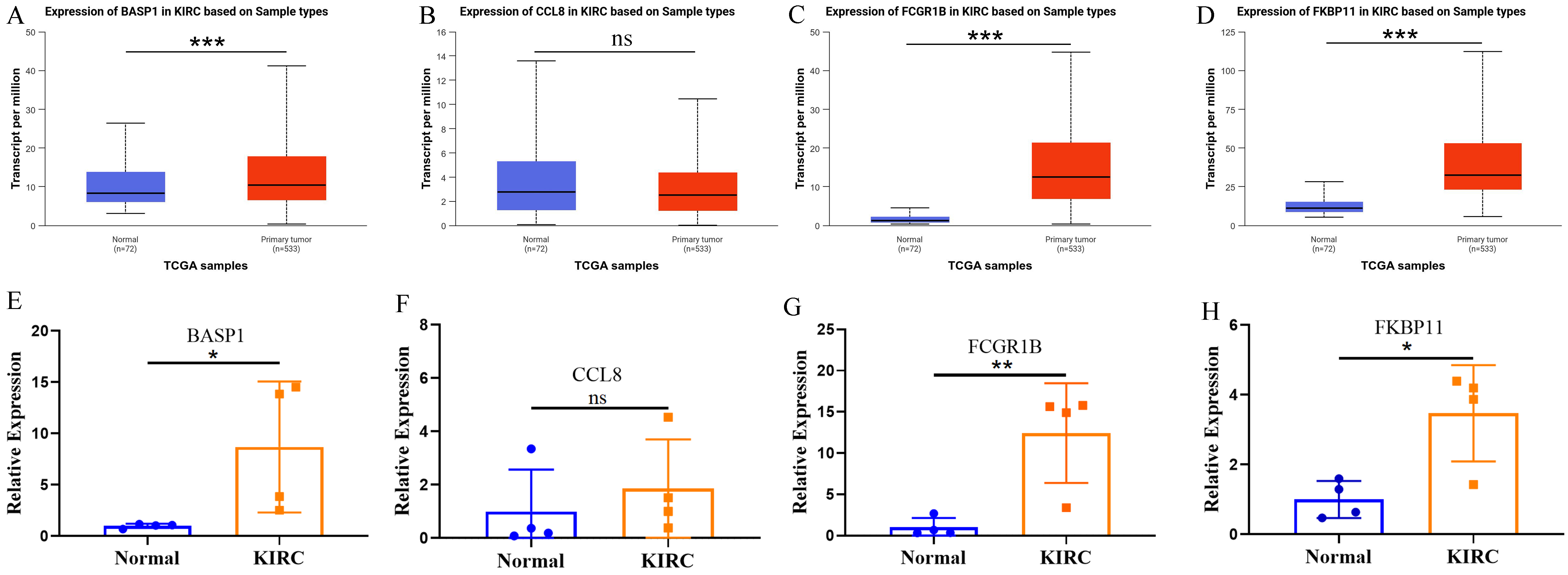

Supplement: Supplementary file 1 [file DataSheet1.zip › all raw data/Figures/Figure/Figure 7.jpg]

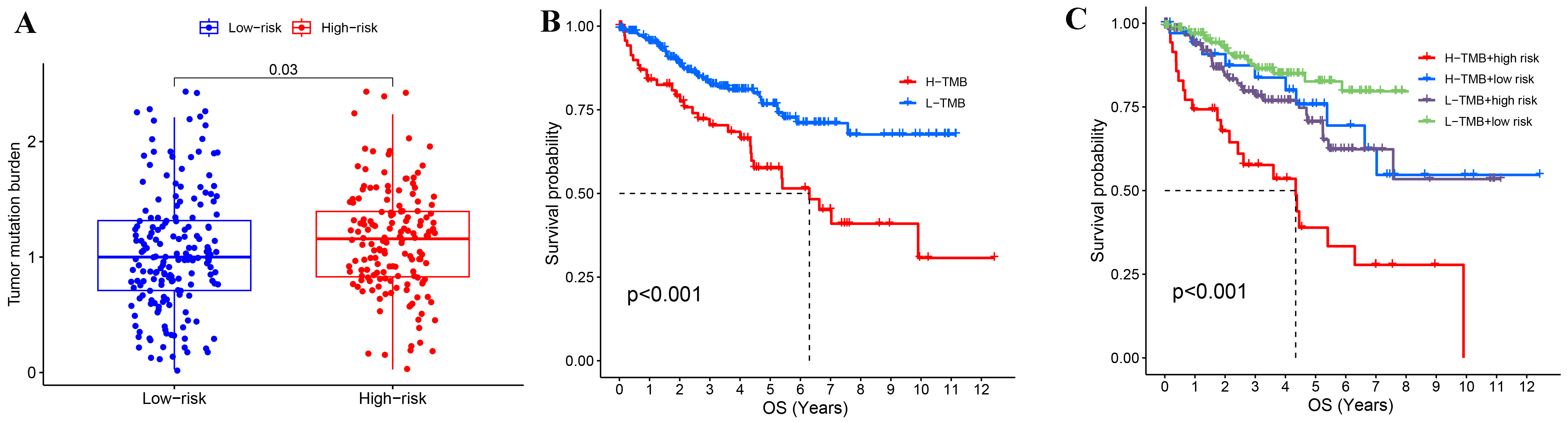

Supplement: Supplementary file 1 [file DataSheet1.zip › all raw data/Figures/Figure/Figure 8.jpg]

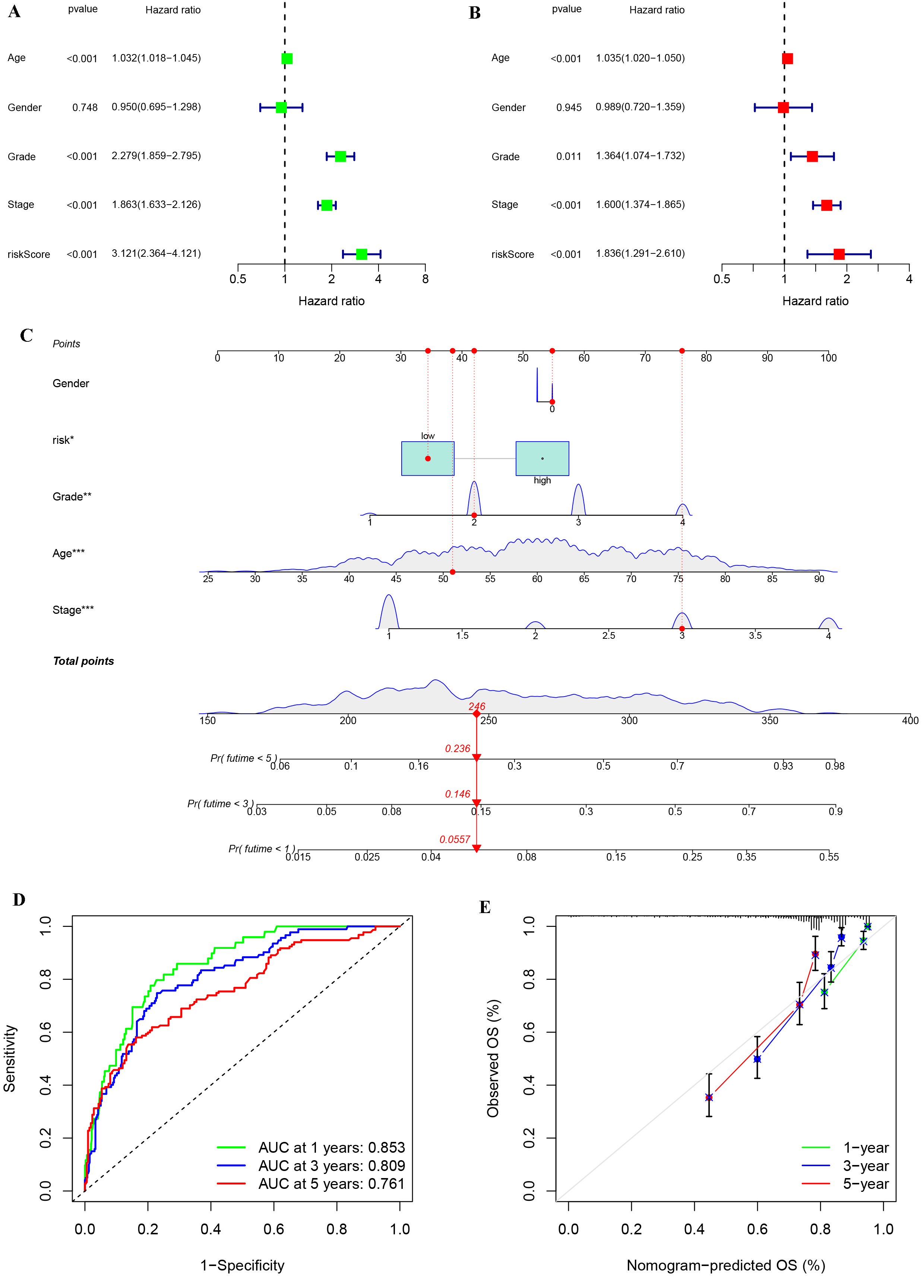

Supplement: Supplementary file 1 [file DataSheet1.zip › all raw data/Figures/Figure/Figure 9.jpg]

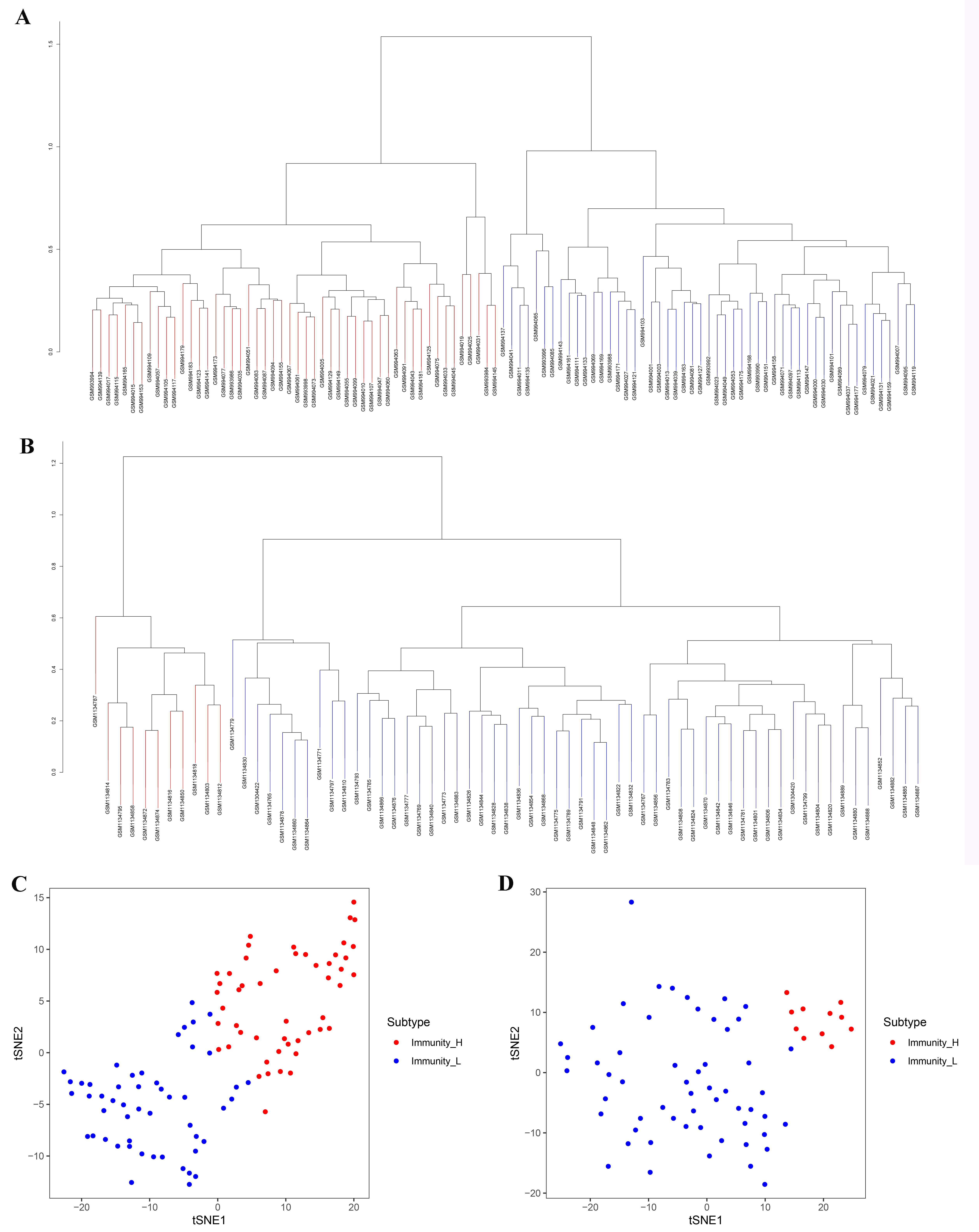

Supplement: Supplementary file 1 [file DataSheet1.zip › all raw data/Figures/Figure 1/Figure 1.jpg]

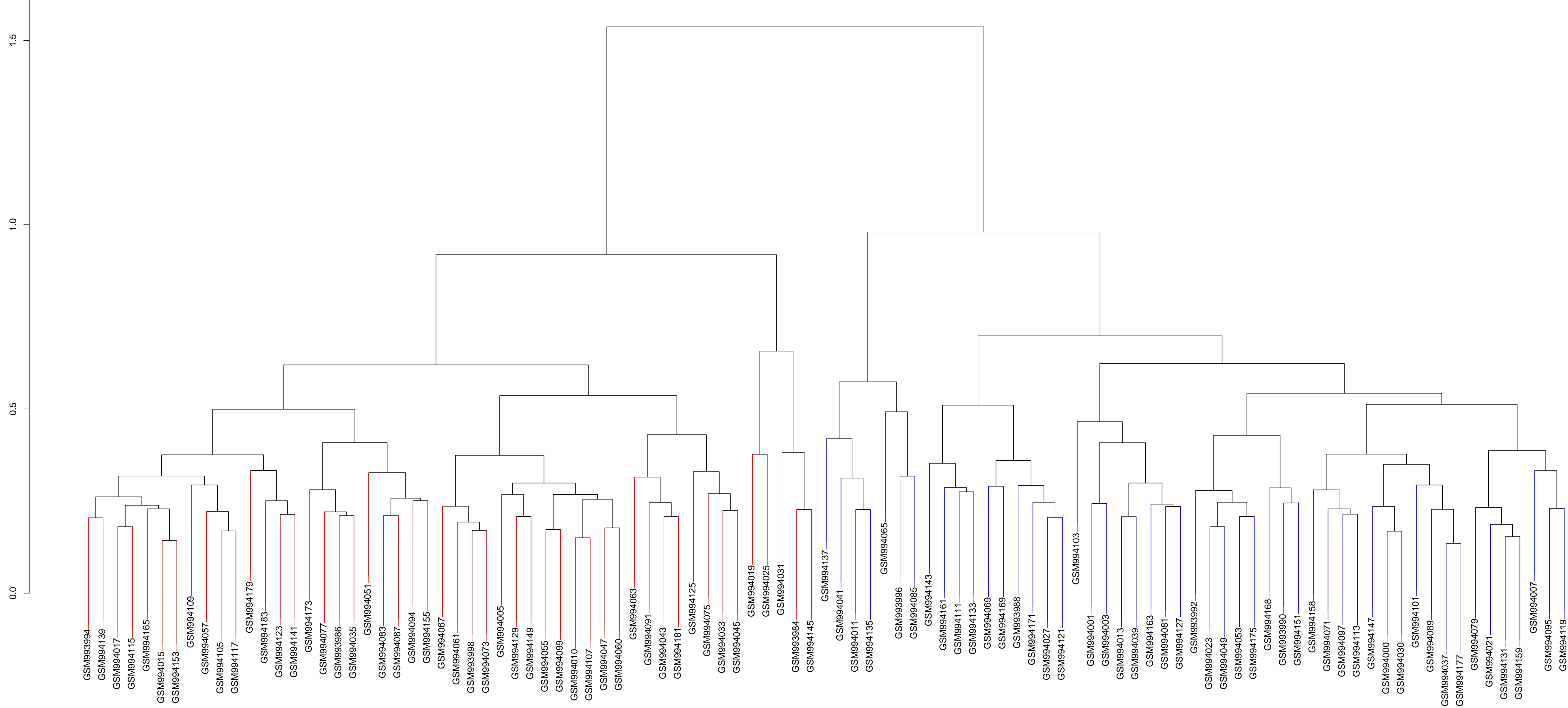

Supplement: Supplementary file 1 [file DataSheet1.zip › all raw data/Figures/Figure 1/Figure 1A.pdf]

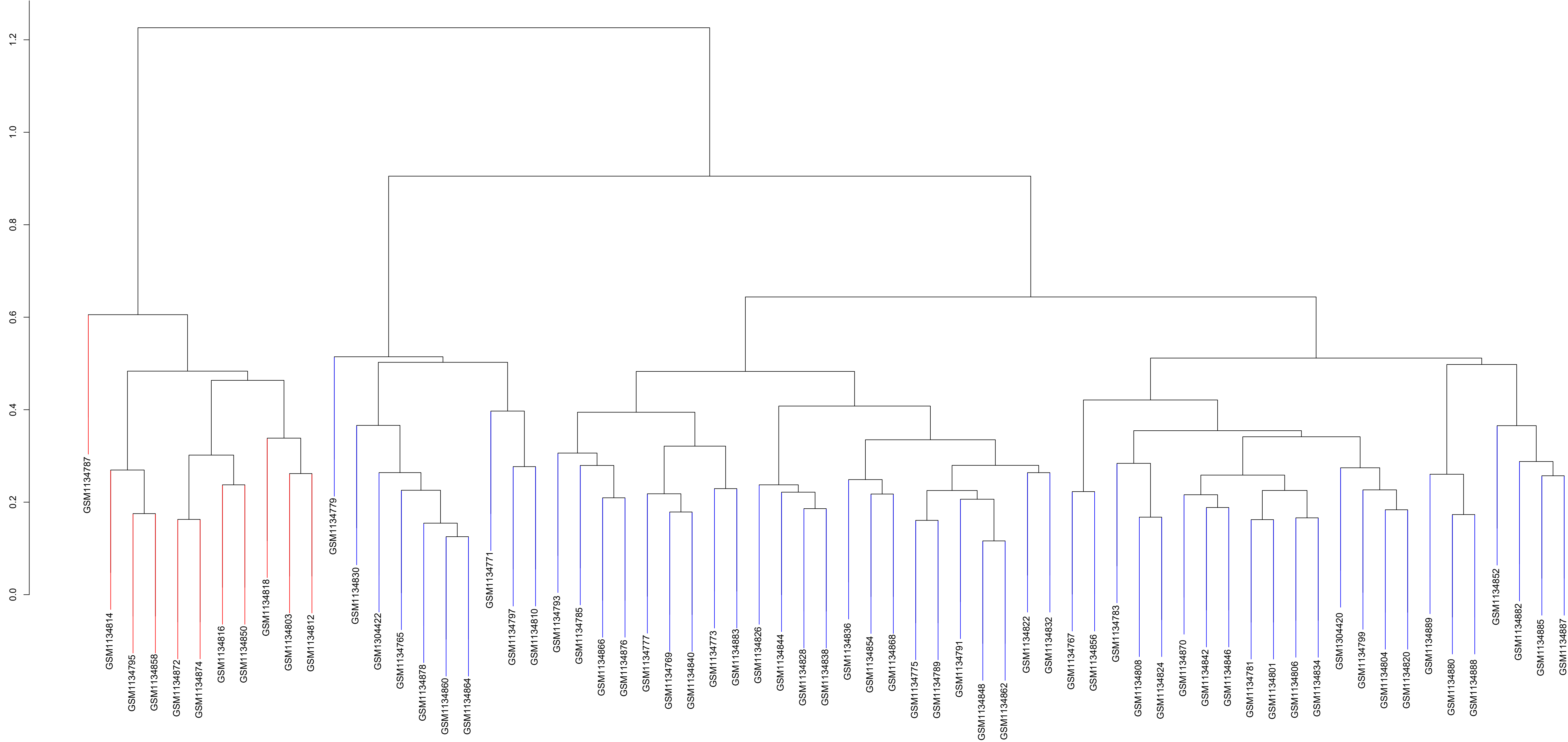

Supplement: Supplementary file 1 [file DataSheet1.zip › all raw data/Figures/Figure 1/Figure 1B.pdf]

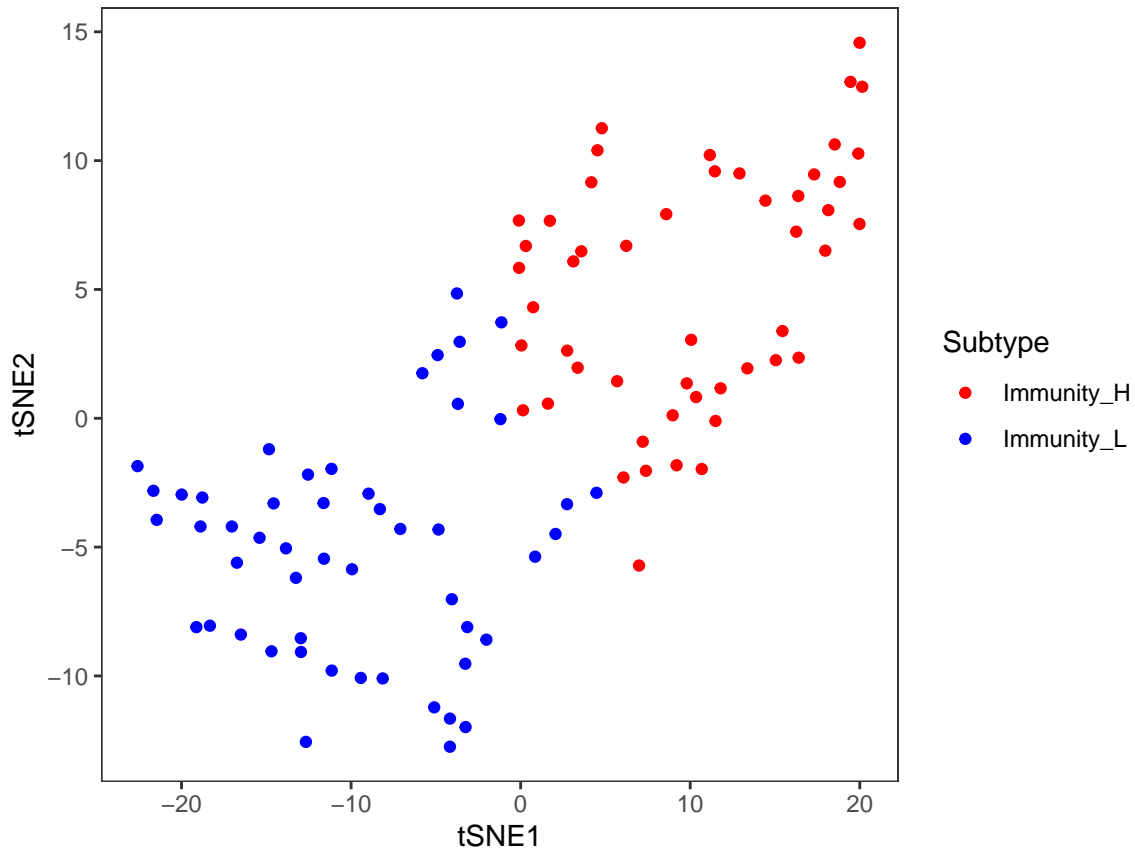

Supplement: Supplementary file 1 [file DataSheet1.zip › all raw data/Figures/Figure 1/Figure 1C.pdf]

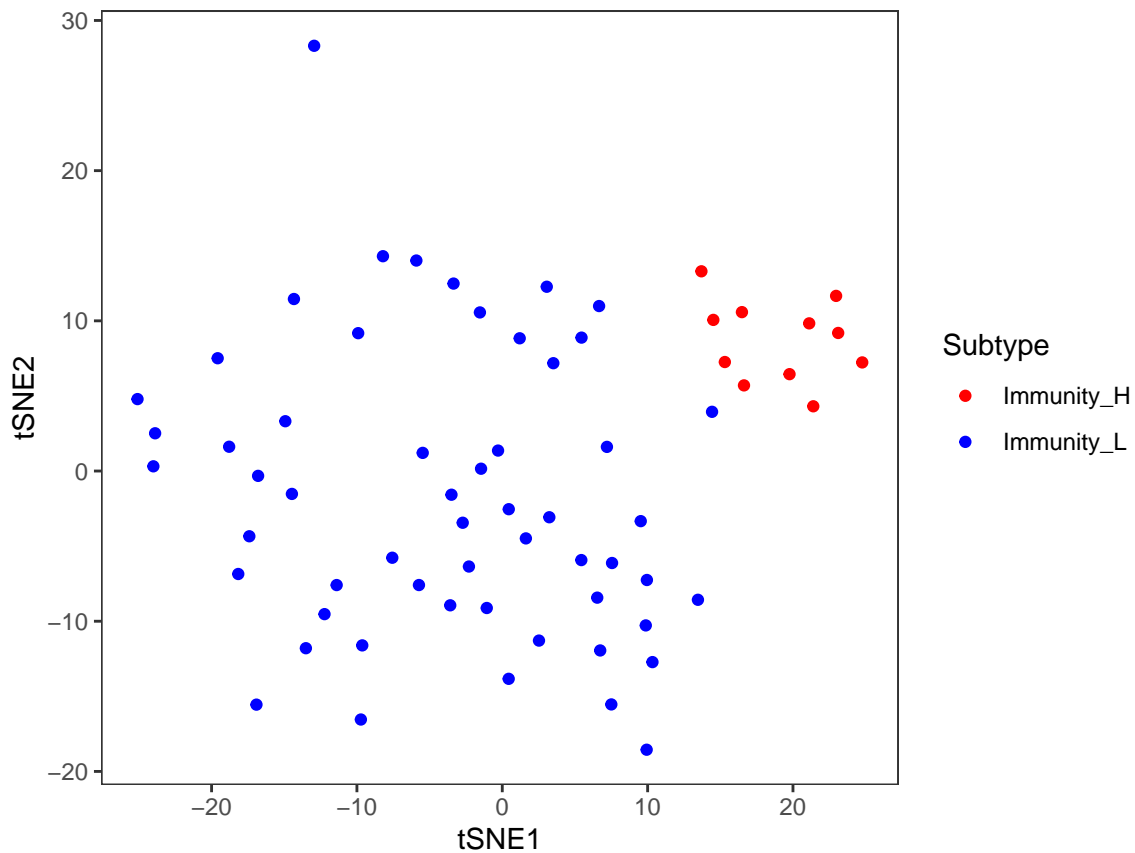

Supplement: Supplementary file 1 [file DataSheet1.zip › all raw data/Figures/Figure 1/Figure 1D.pdf]

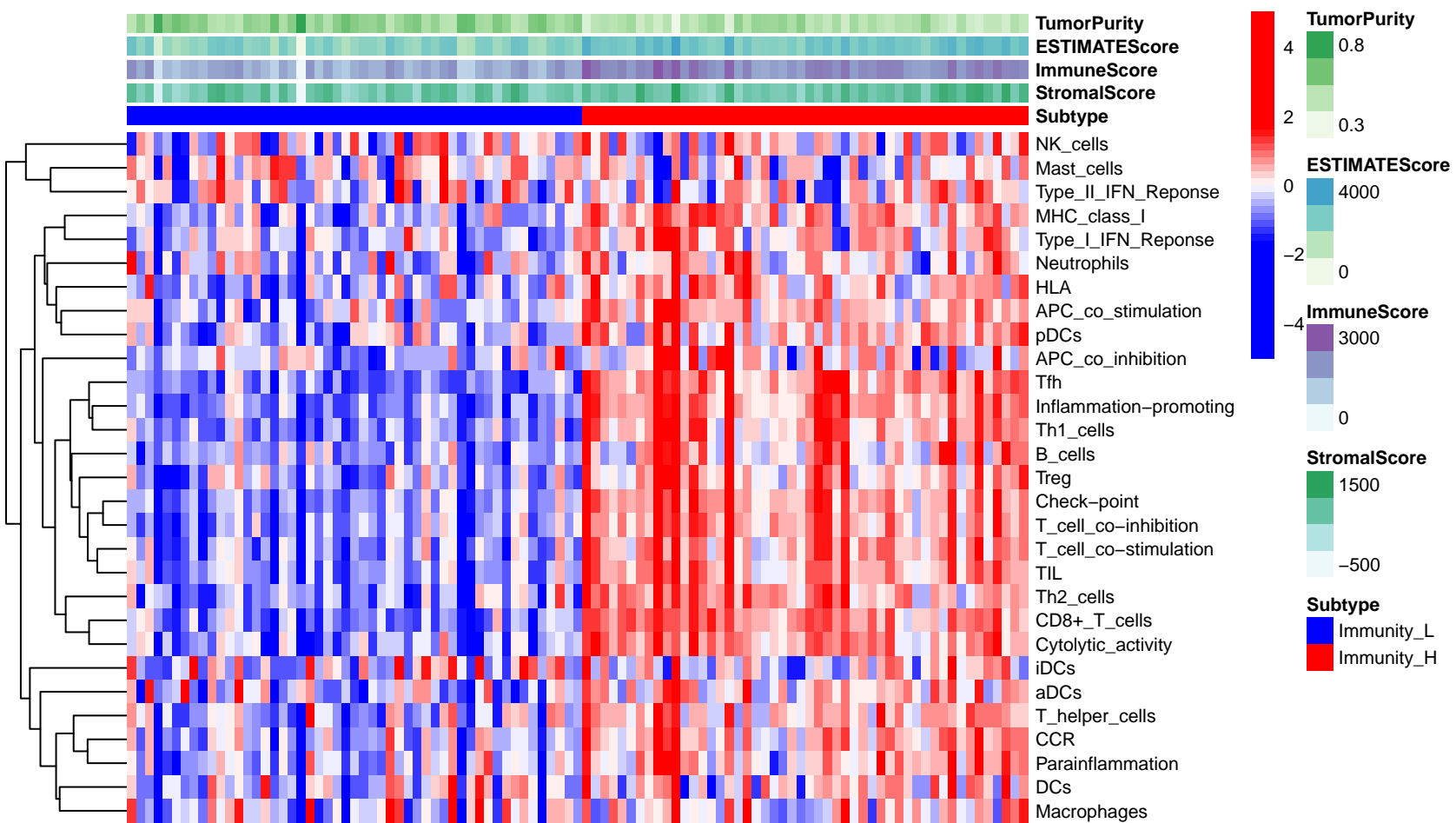

Supplement: Supplementary file 1 [file DataSheet1.zip › all raw data/Figures/Figure 2/Figure 2A.pdf]

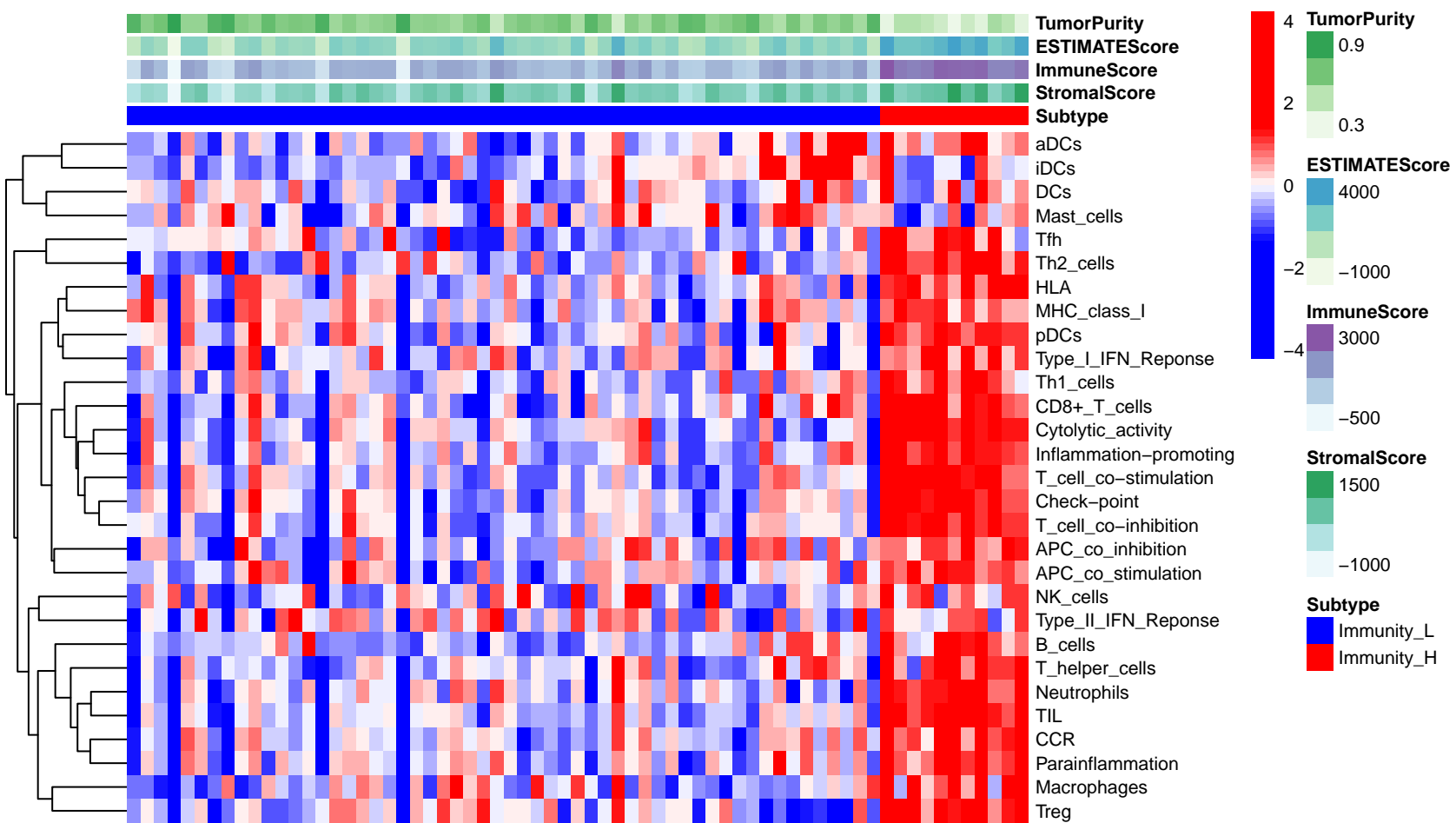

Supplement: Supplementary file 1 [file DataSheet1.zip › all raw data/Figures/Figure 2/Figure 2B.pdf]

Subtype 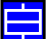 Immunity\_L 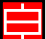 Immunity\_H

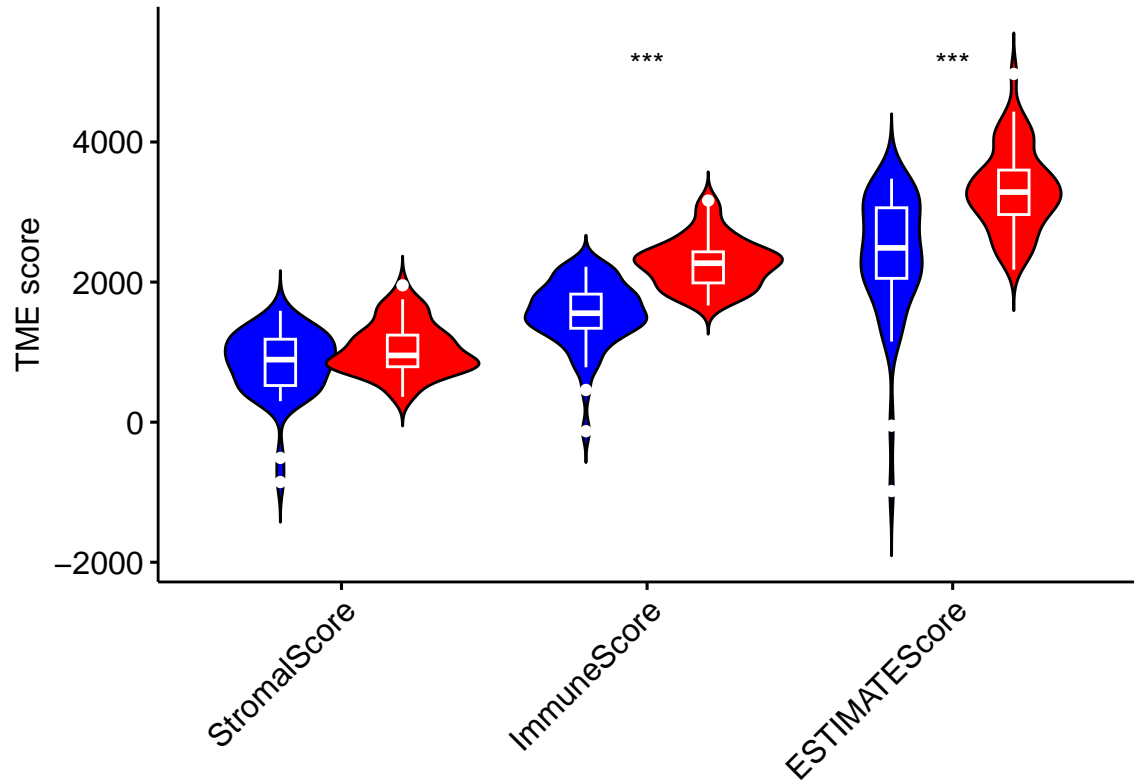

Supplement: Supplementary file 1 [file DataSheet1.zip › all raw data/Figures/Figure 2/Figure 2C.pdf]

Subtype

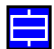

Immunity\_L

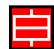

Immunity\_H

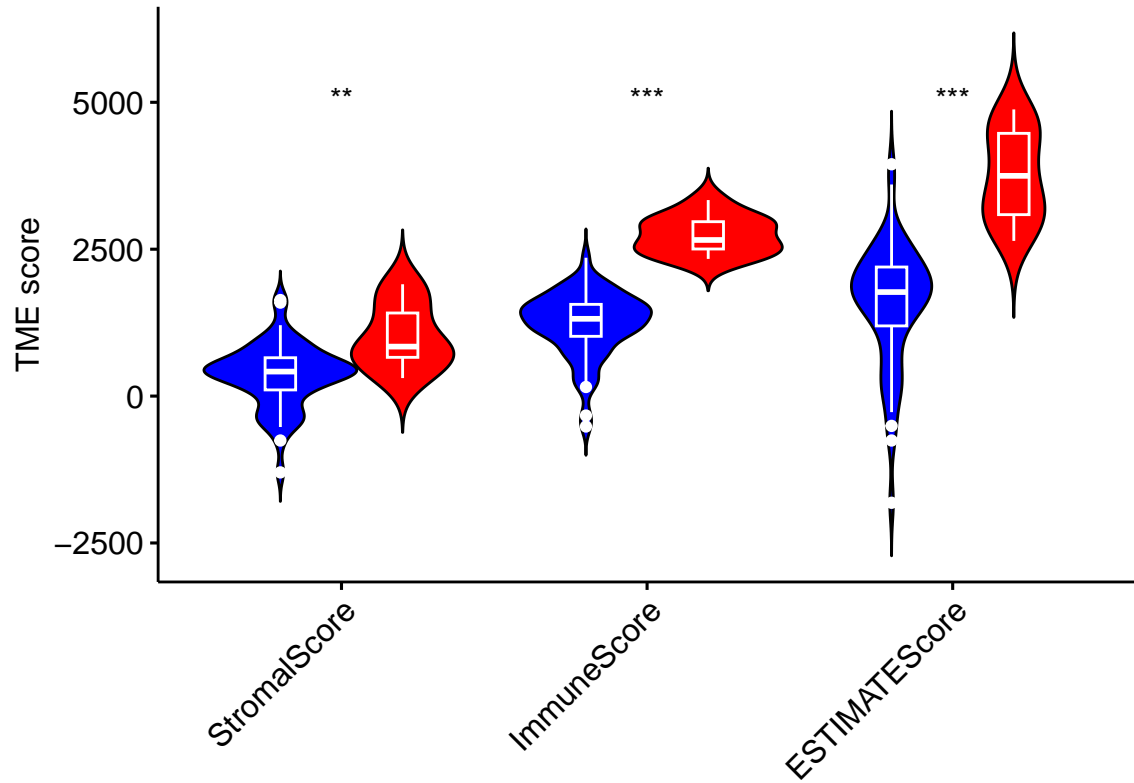

Supplement: Supplementary file 1 [file DataSheet1.zip › all raw data/Figures/Figure 2/Figure 2D.pdf]

Subtype Immunity\_L Immunity\_H

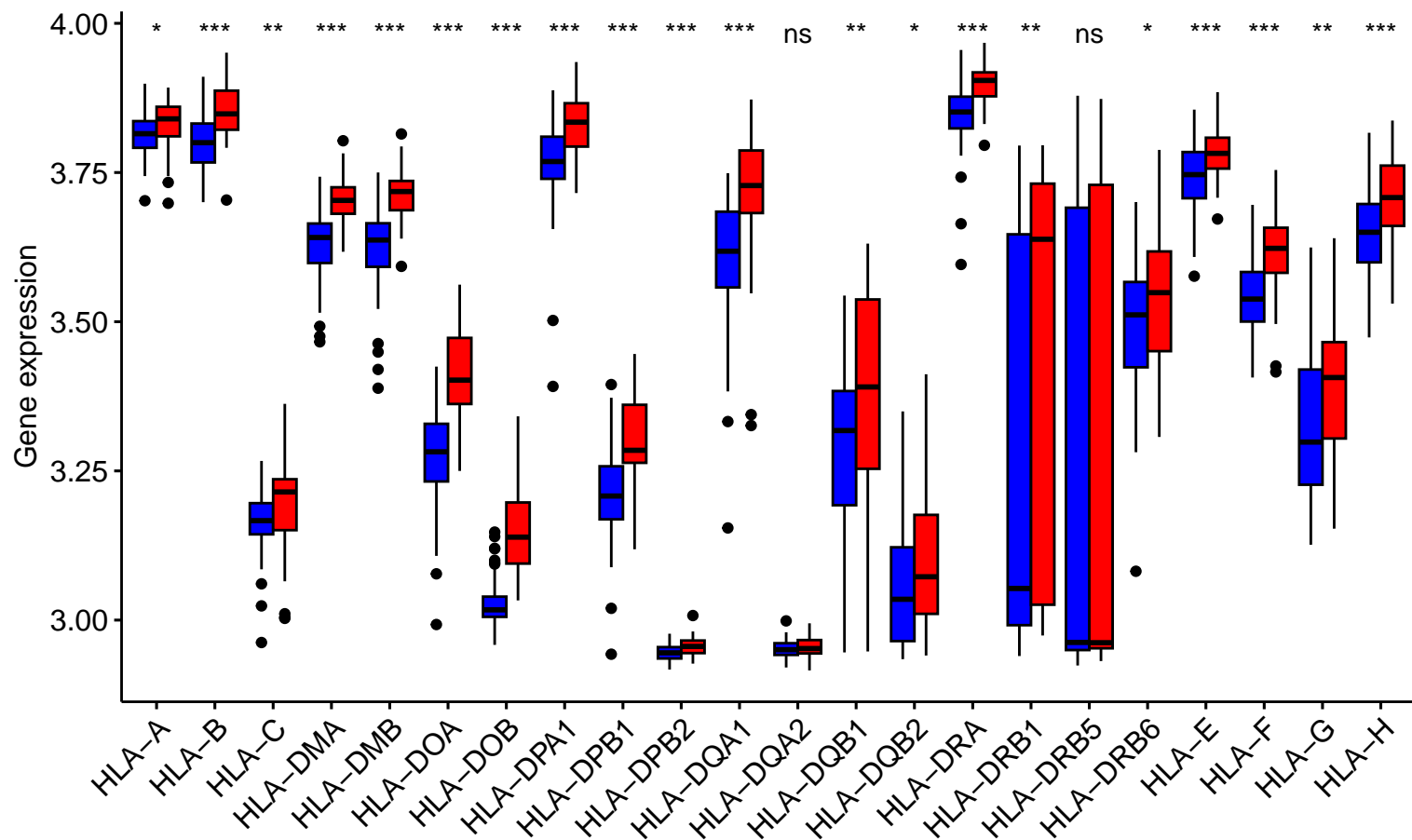

Supplement: Supplementary file 1 [file DataSheet1.zip › all raw data/Figures/Figure 2/Figure 2E.pdf]

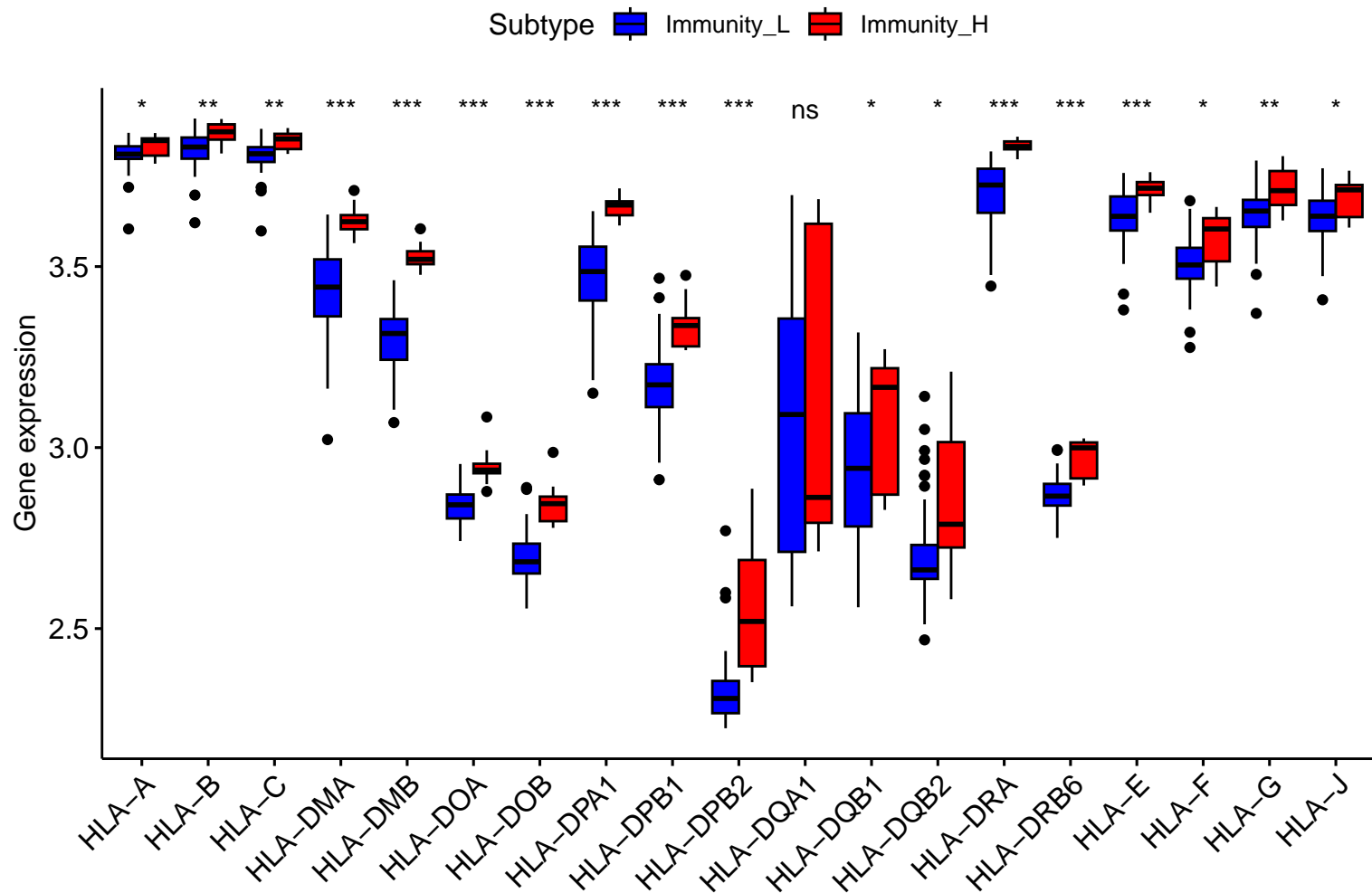

Supplement: Supplementary file 1 [file DataSheet1.zip › all raw data/Figures/Figure 2/Figure 2F.pdf]

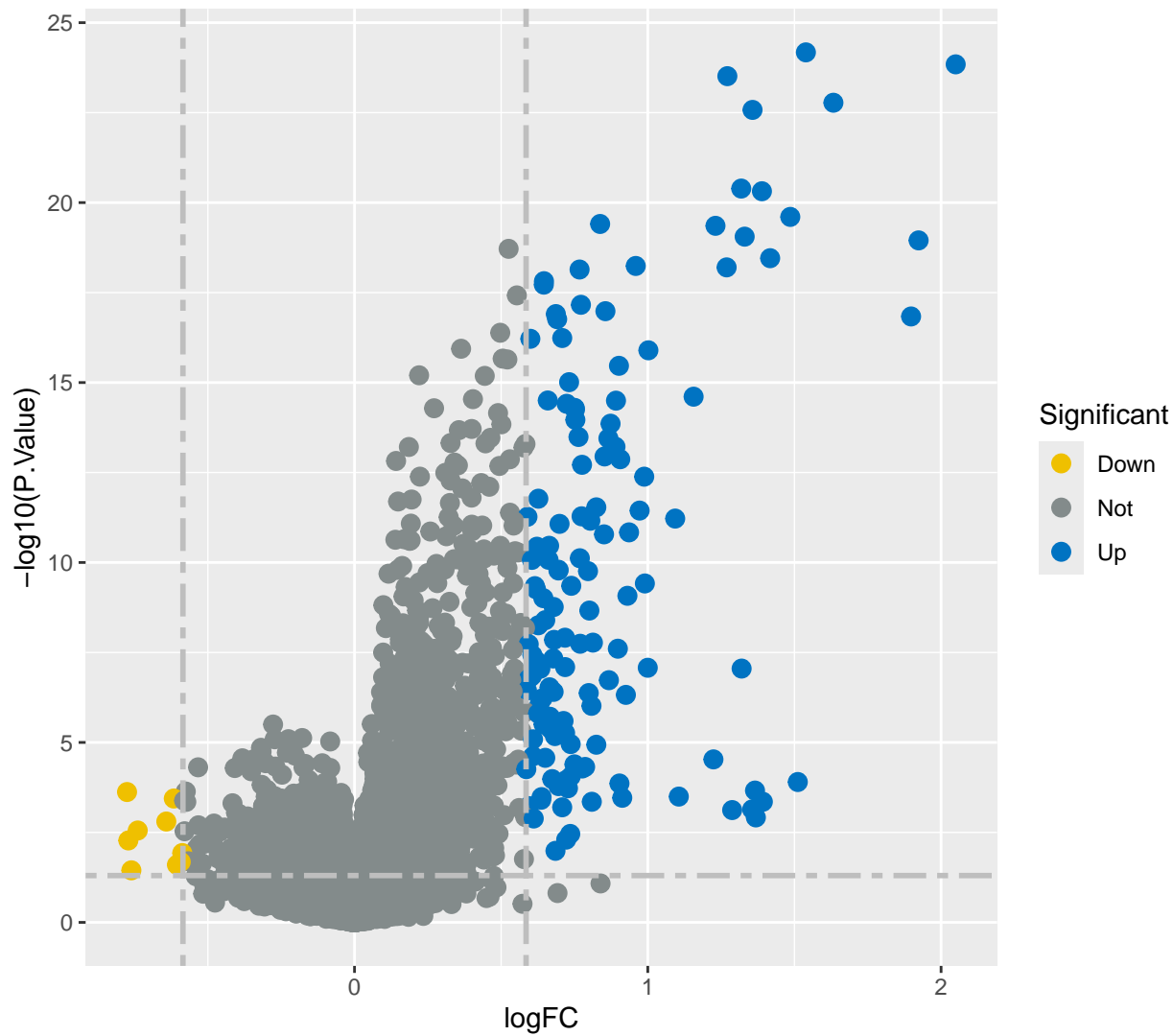

Supplement: Supplementary file 1 [file DataSheet1.zip › all raw data/Figures/Figure 3/Figure 3A.pdf]

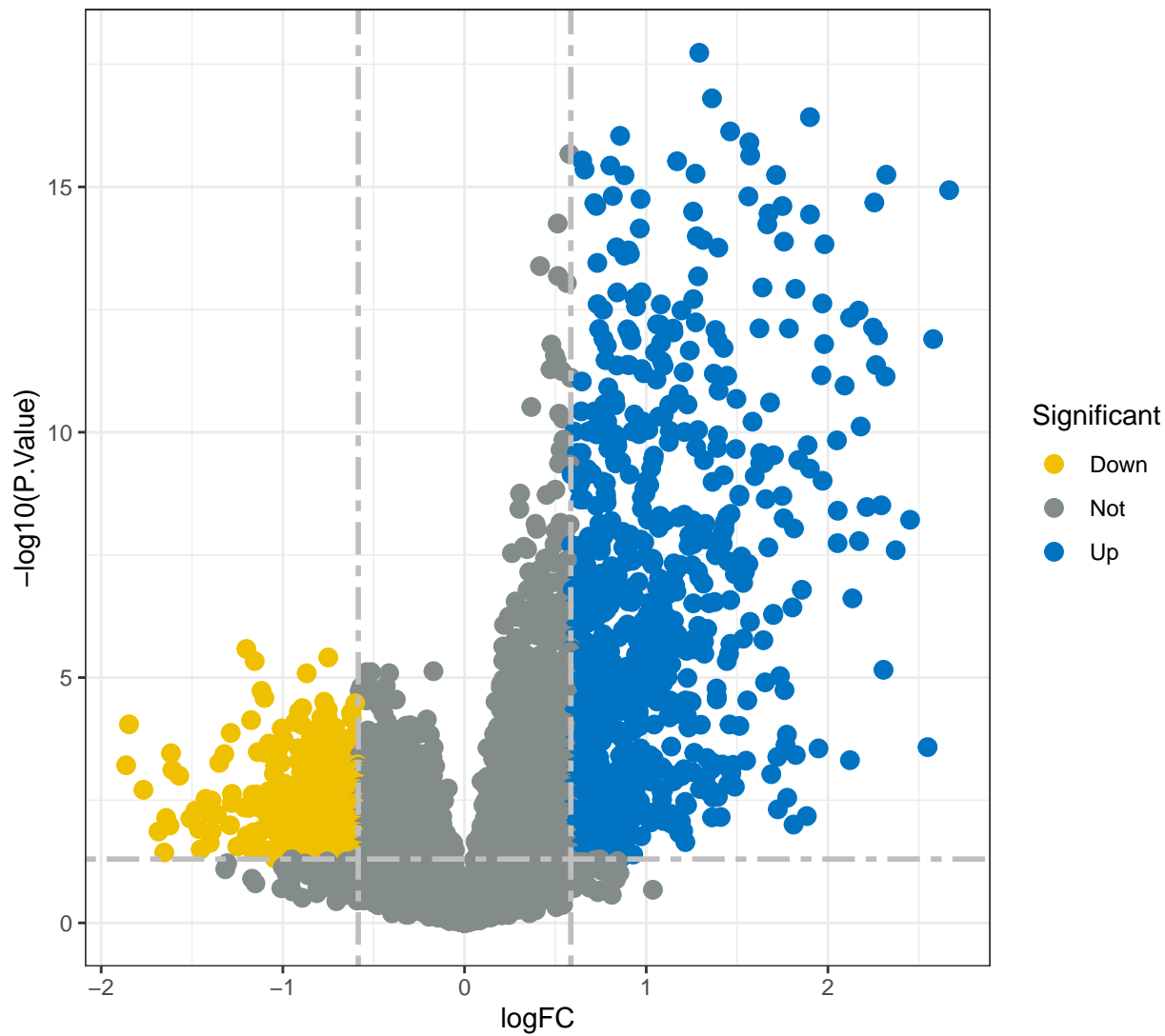

Supplement: Supplementary file 1 [file DataSheet1.zip › all raw data/Figures/Figure 3/Figure 3B.pdf]

GSE40435\_UP

GSE46699\_UP

**38**

**100**

**721**

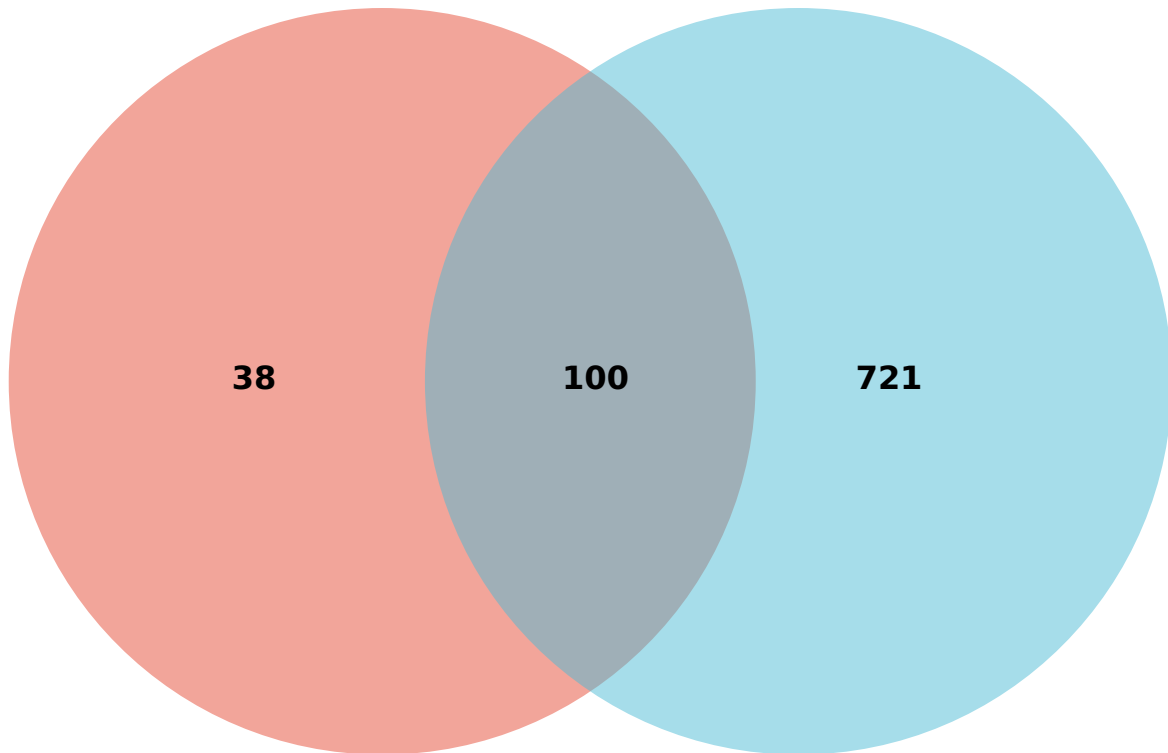

Supplement: Supplementary file 1 [file DataSheet1.zip › all raw data/Figures/Figure 3/Figure 3C.pdf]

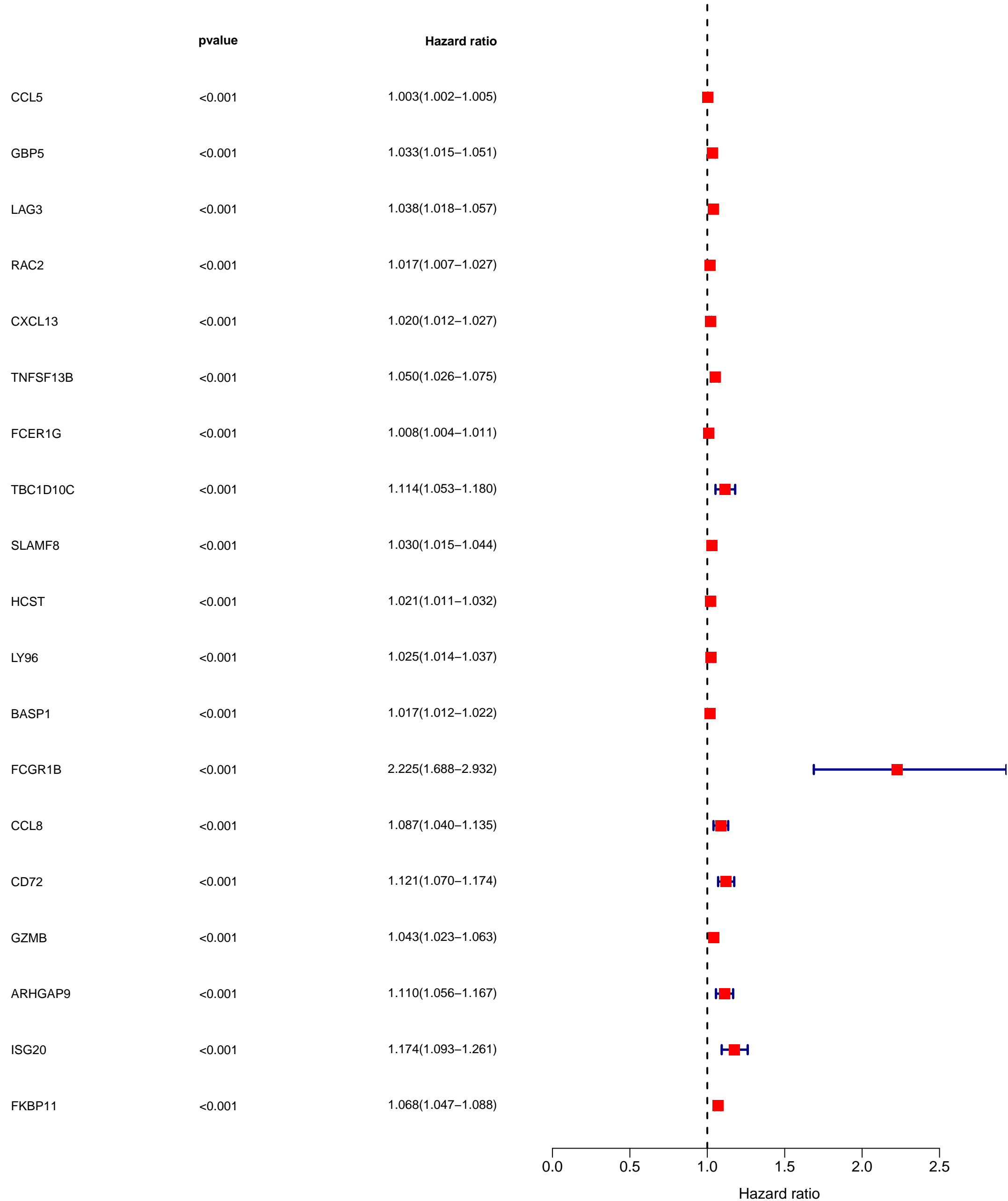

Supplement: Supplementary file 1 [file DataSheet1.zip › all raw data/Figures/Figure 4/Figure 4A.pdf]

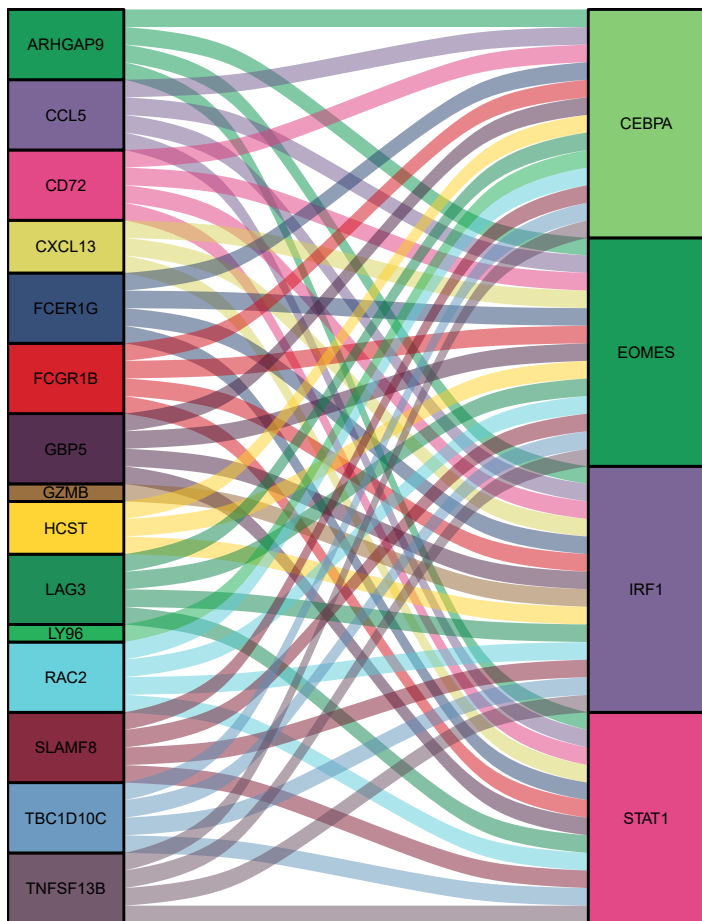

PRGs

TFs

Supplement: Supplementary file 1 [file DataSheet1.zip › all raw data/Figures/Figure 4/Figure 4B.pdf]

Partial Likelihood Deviance

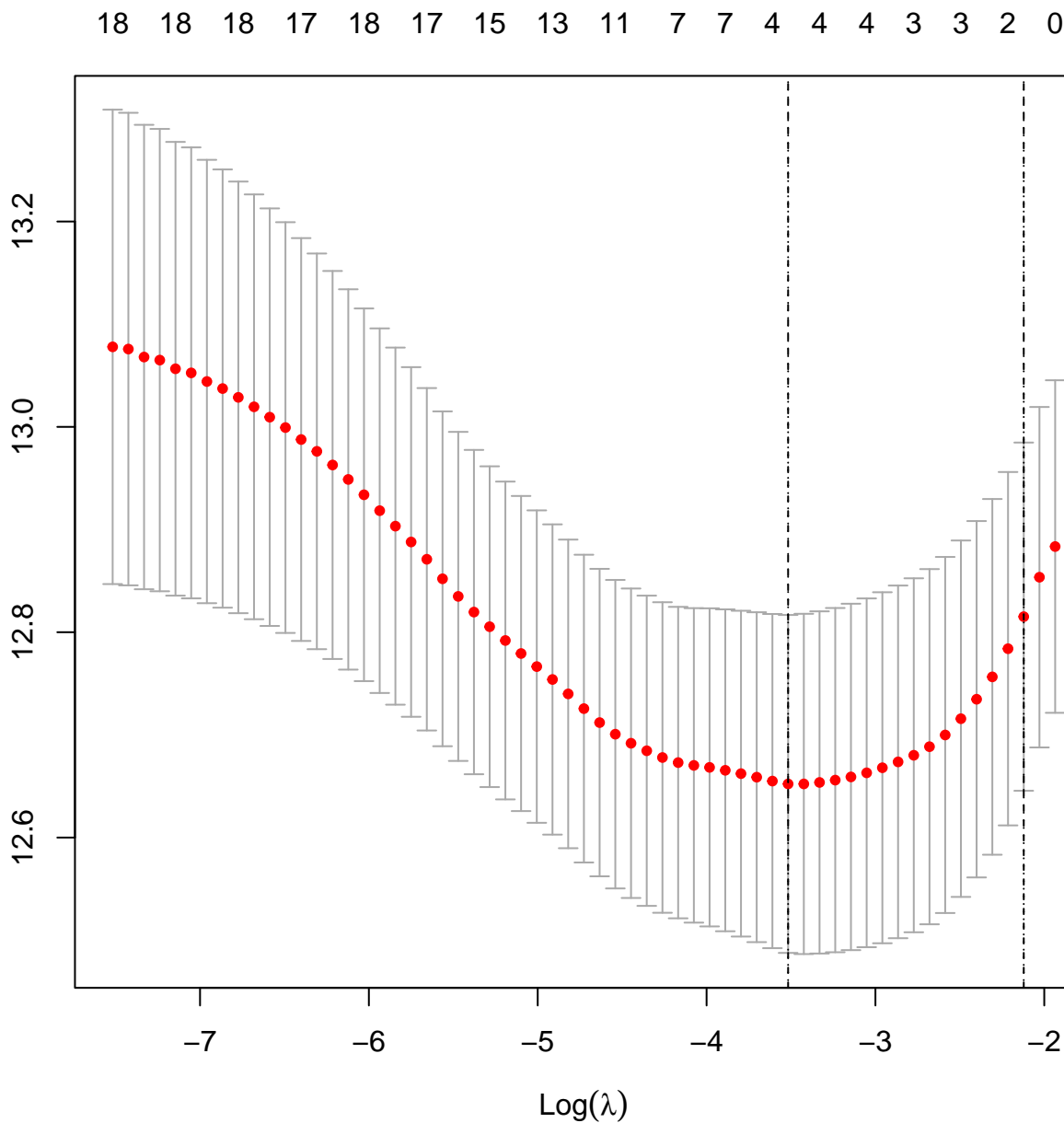

Supplement: Supplementary file 1 [file DataSheet1.zip › all raw data/Figures/Figure 5/Figure 5A.pdf]

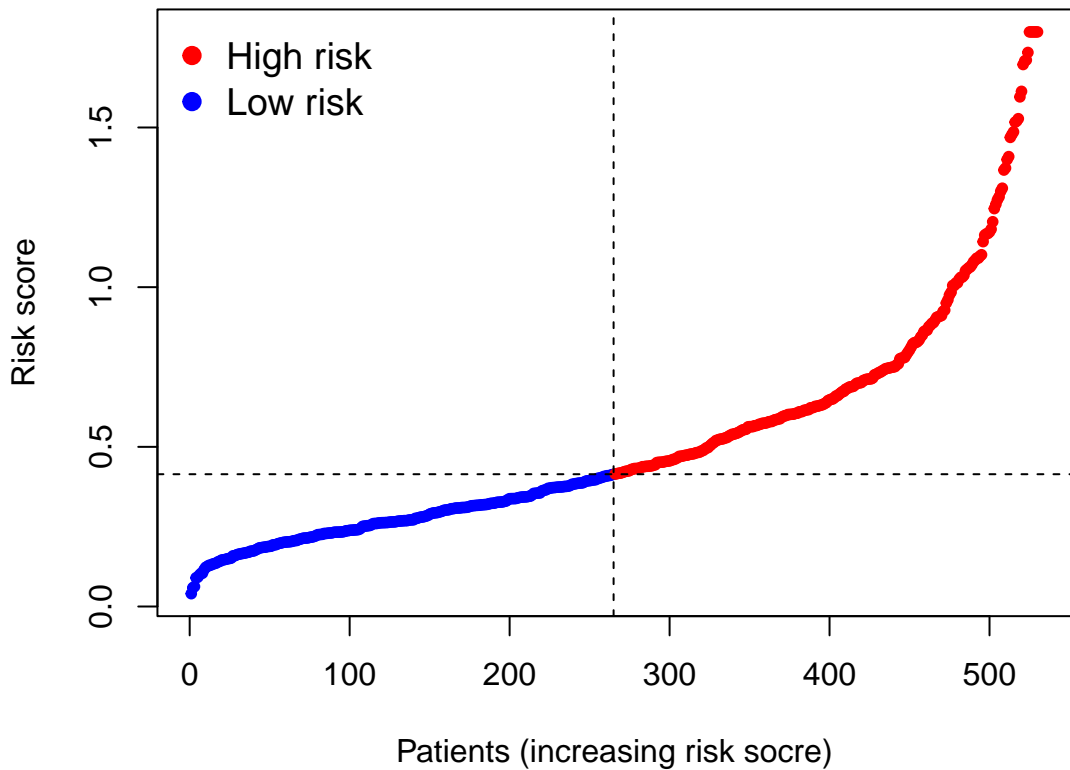

Supplement: Supplementary file 1 [file DataSheet1.zip › all raw data/Figures/Figure 5/Figure 5B.pdf]

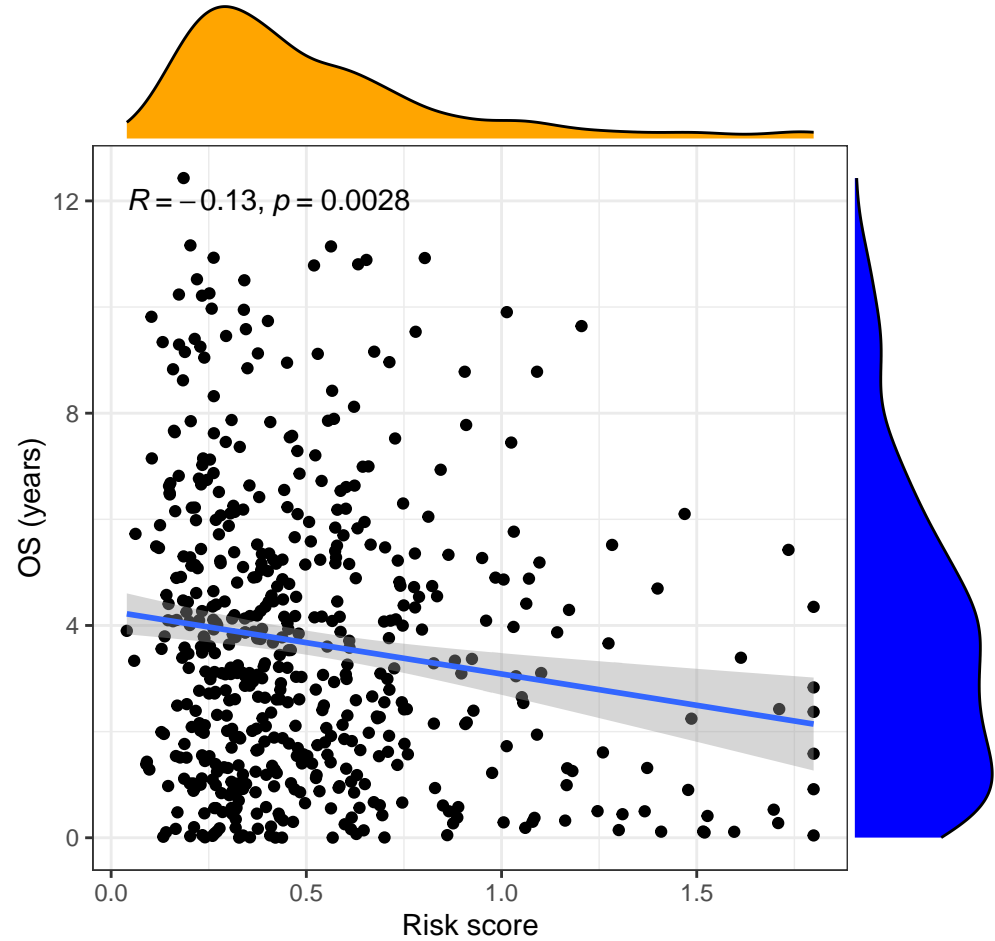

Supplement: Supplementary file 1 [file DataSheet1.zip › all raw data/Figures/Figure 5/Figure 5C.pdf]

Risk High risk Low risk

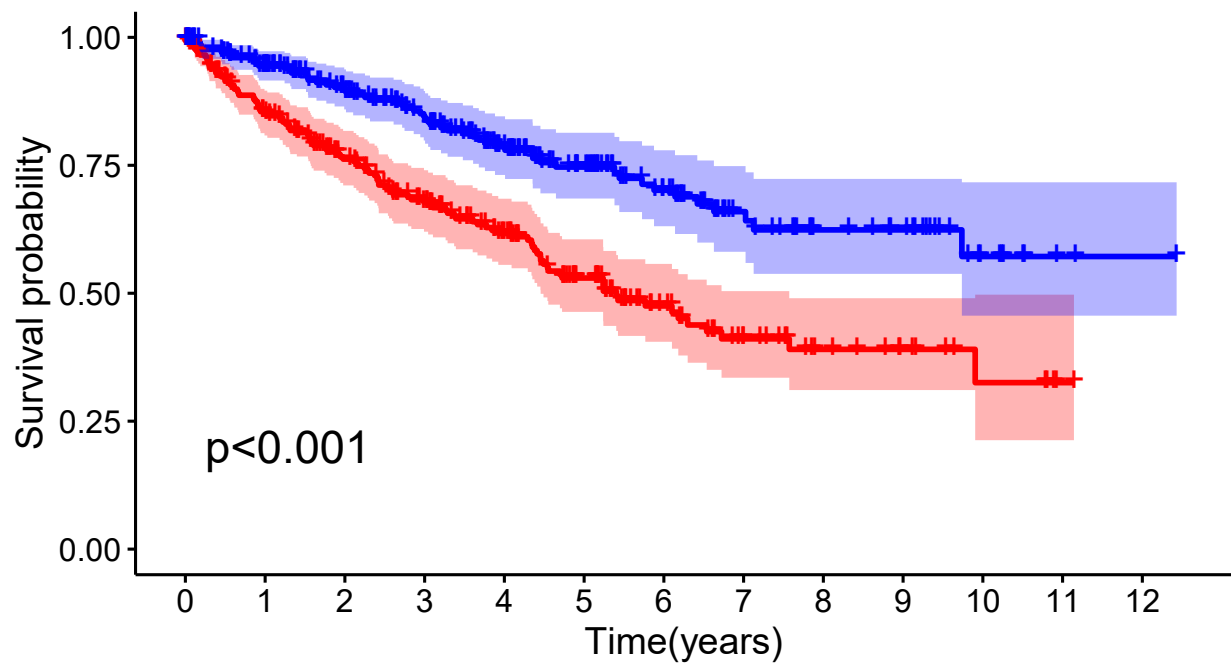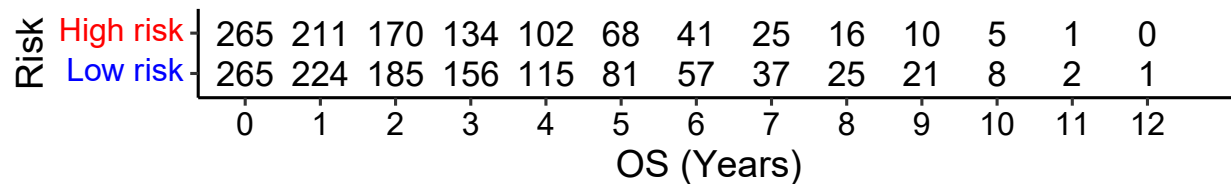

Supplement: Supplementary file 1 [file DataSheet1.zip › all raw data/Figures/Figure 5/Figure 5D.pdf]

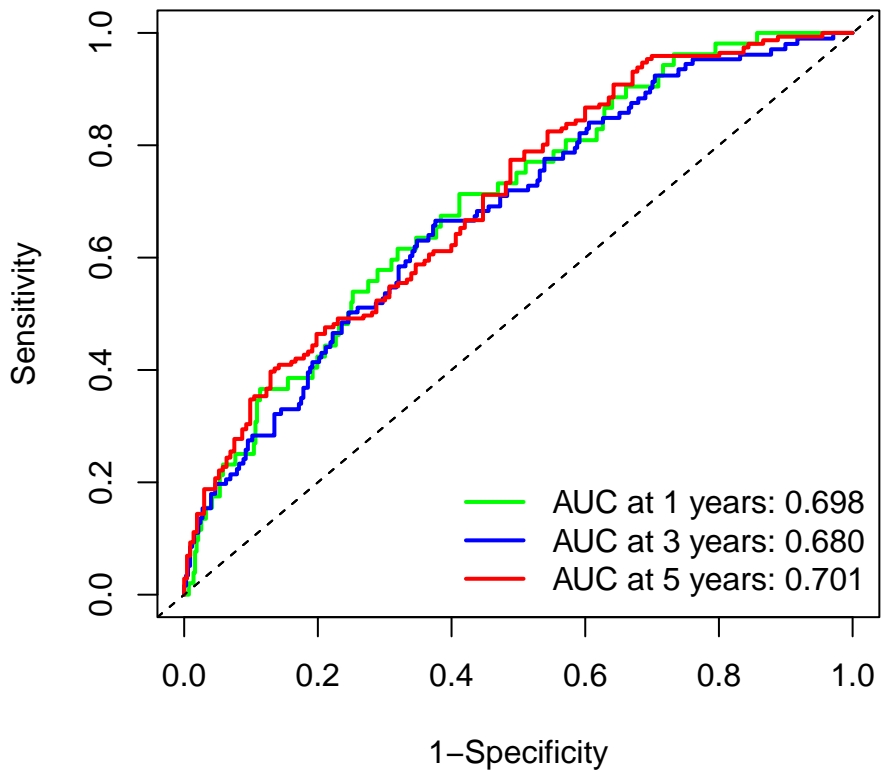

Supplement: Supplementary file 1 [file DataSheet1.zip › all raw data/Figures/Figure 5/Figure 5E.pdf]

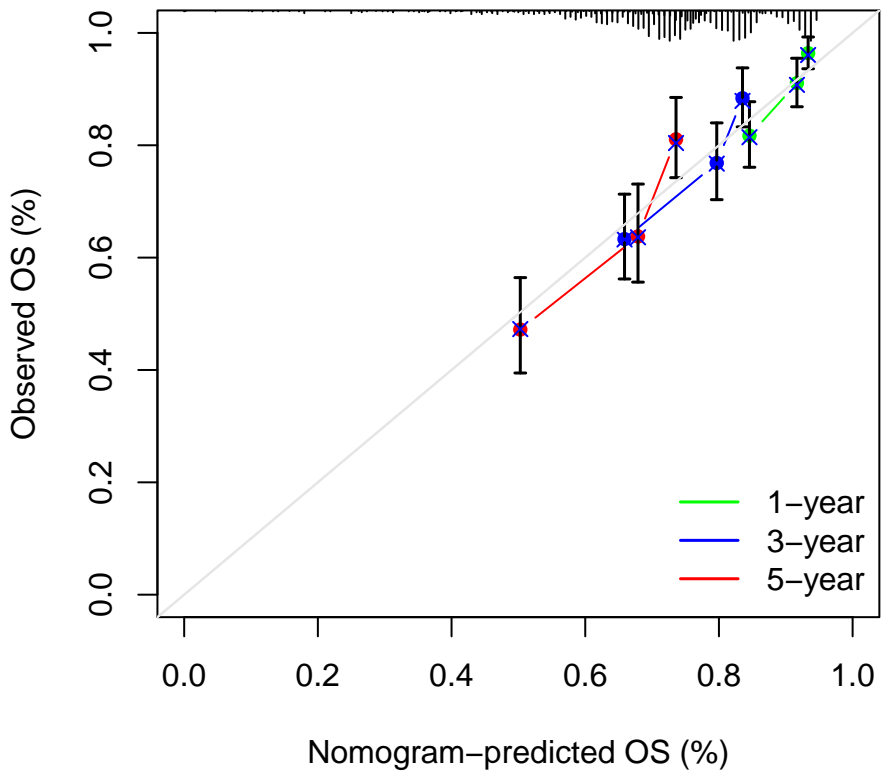

Supplement: Supplementary file 1 [file DataSheet1.zip › all raw data/Figures/Figure 5/Figure 5F.pdf]

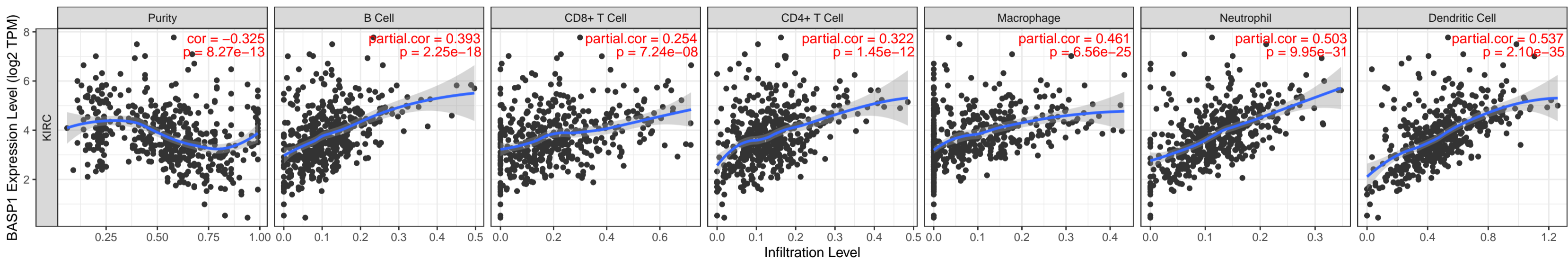

Supplement: Supplementary file 1 [file DataSheet1.zip › all raw data/Figures/Figure 6/Figure 6A.pdf]

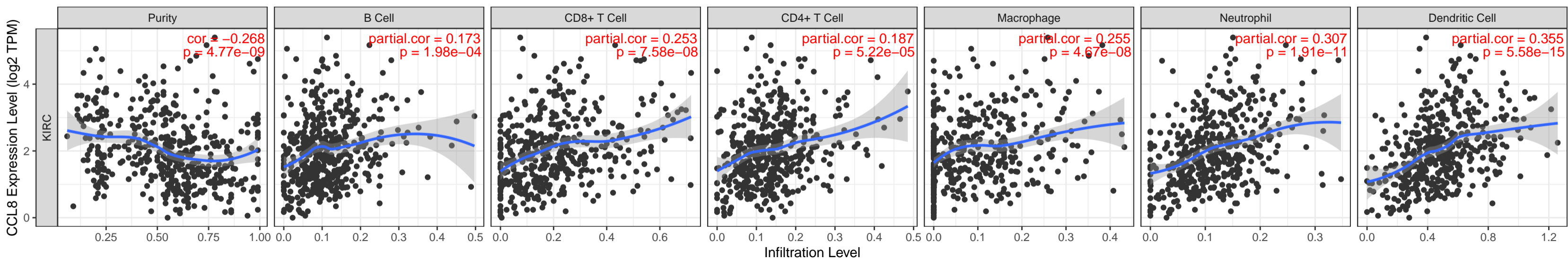

Supplement: Supplementary file 1 [file DataSheet1.zip › all raw data/Figures/Figure 6/Figure 6B.pdf]

FCGR1B Expression Level (log2 TPM)

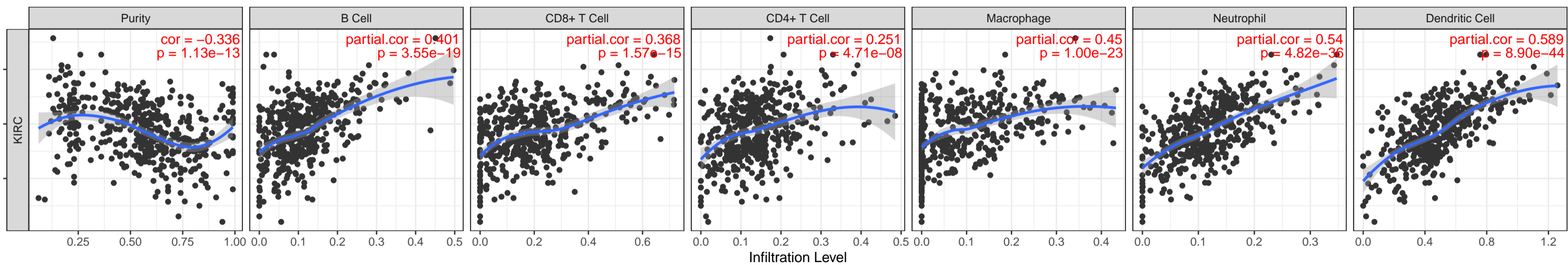

Supplement: Supplementary file 1 [file DataSheet1.zip › all raw data/Figures/Figure 6/Figure 6C.pdf]

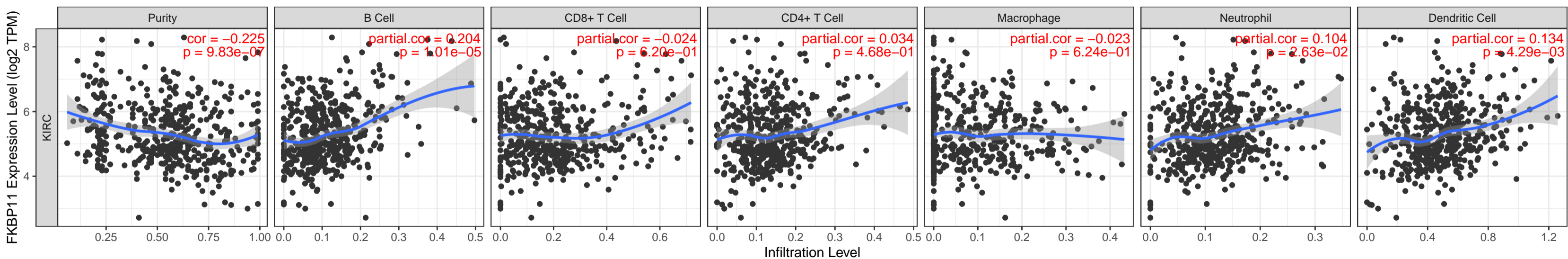

Supplement: Supplementary file 1 [file DataSheet1.zip › all raw data/Figures/Figure 6/Figure 6D.pdf]

# Effect of BASP1 expression level on KIRC patient survival

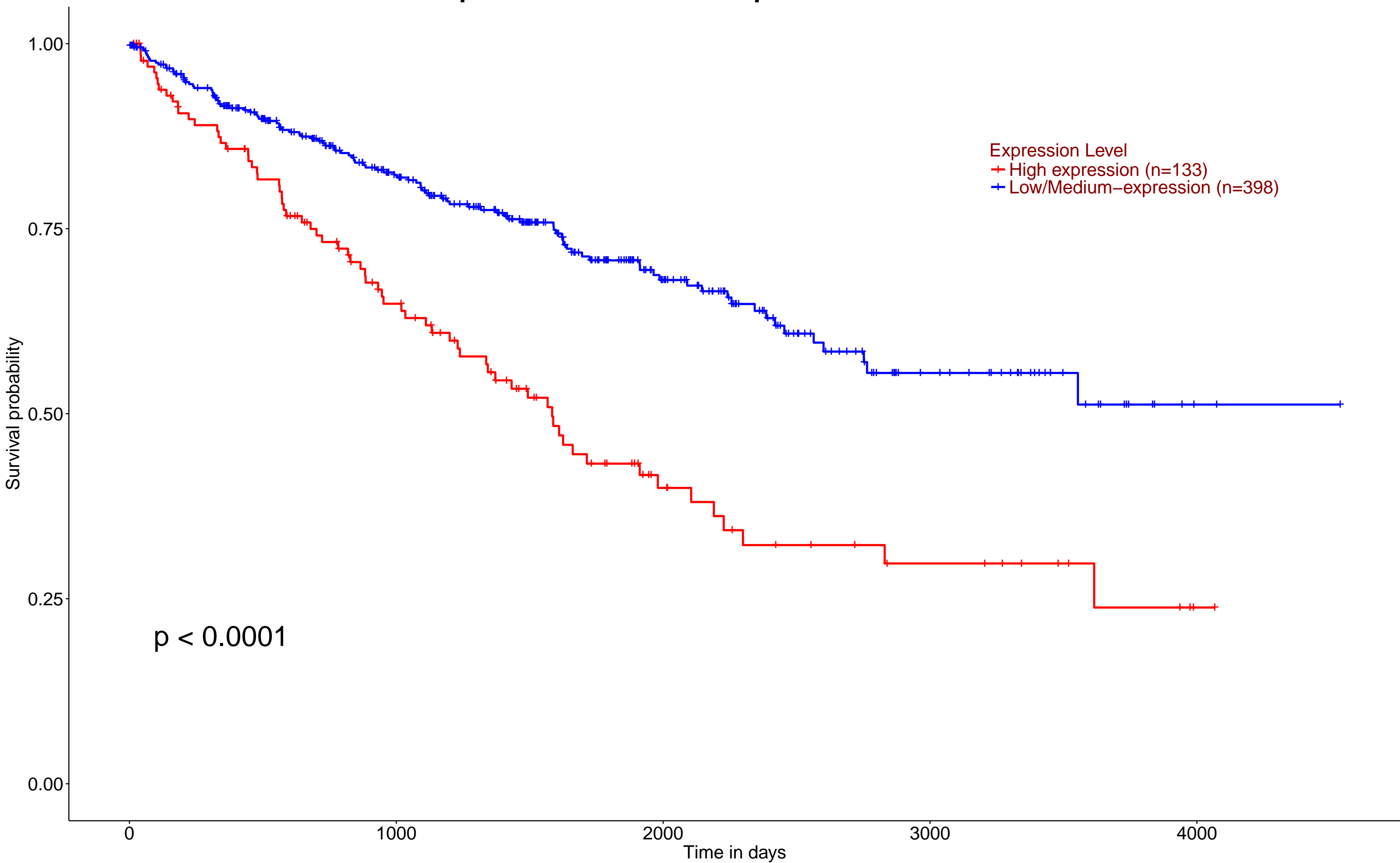

Supplement: Supplementary file 1 [file DataSheet1.zip › all raw data/Figures/Figure 6/Figure 6E.pdf]

# Effect of CCL8 expression level on KIRC patient survival

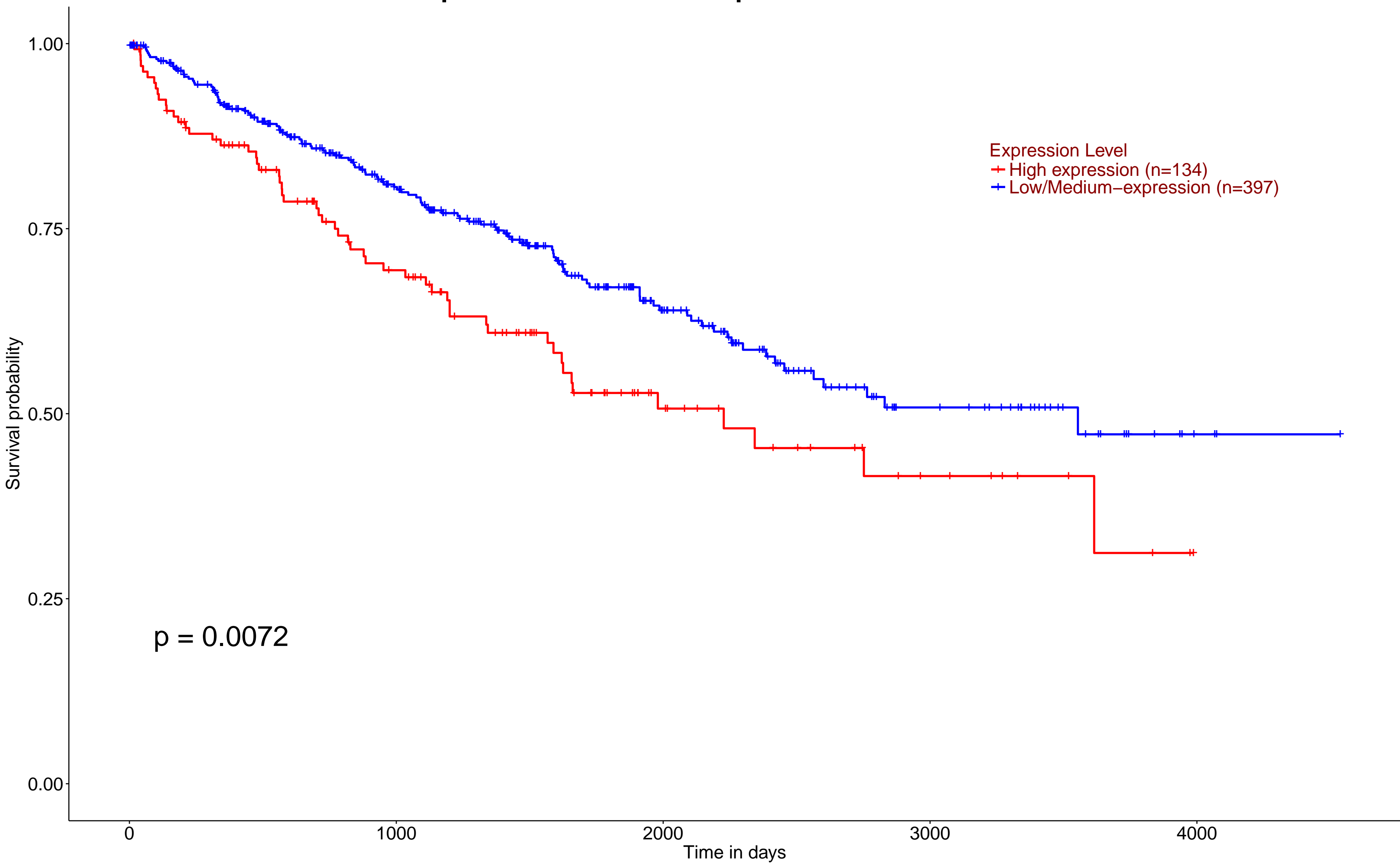

Supplement: Supplementary file 1 [file DataSheet1.zip › all raw data/Figures/Figure 6/Figure 6F.pdf]

# Effect of FCGR1B expression level on KIRC patient survival

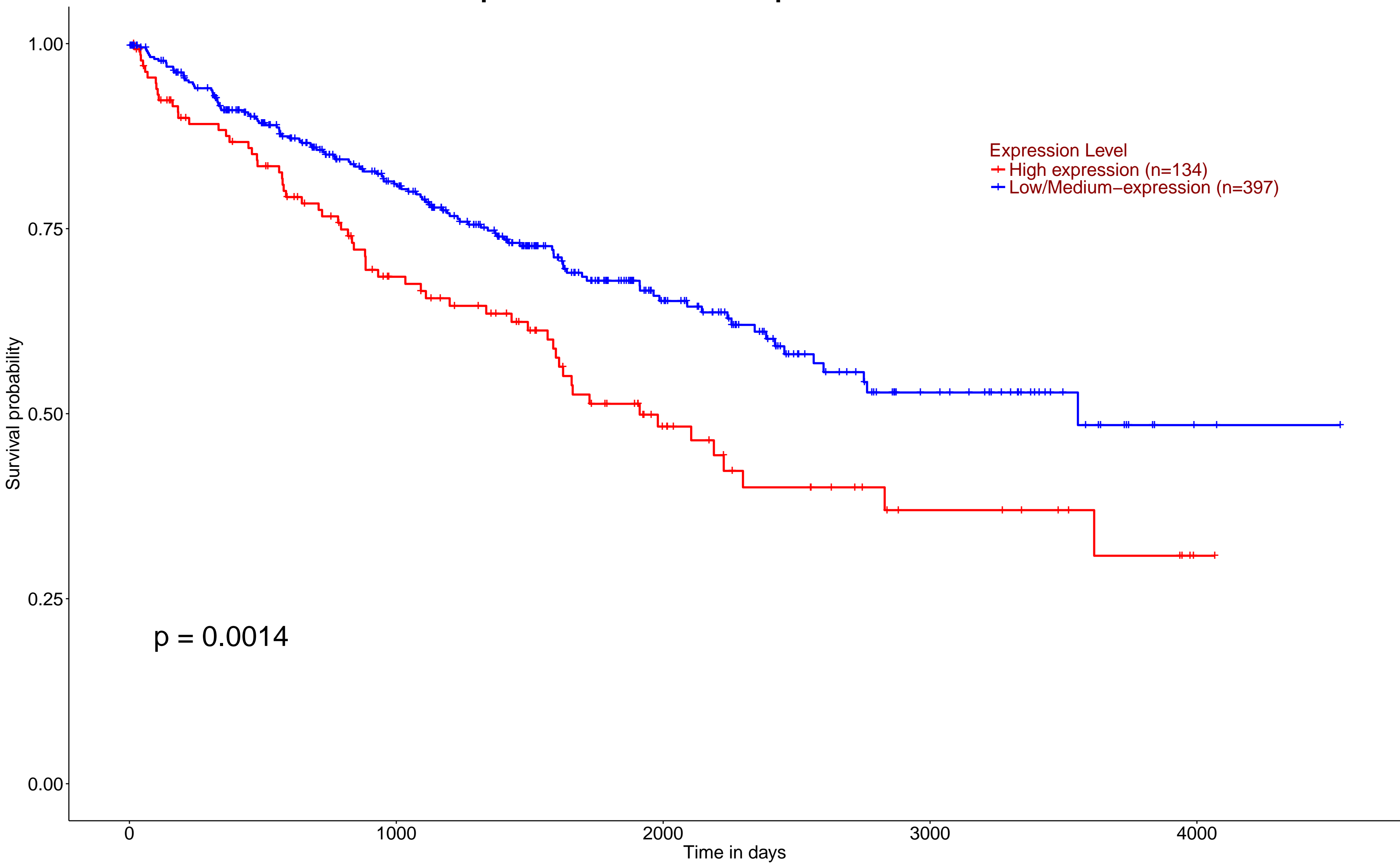

Supplement: Supplementary file 1 [file DataSheet1.zip › all raw data/Figures/Figure 6/Figure 6G.pdf]

Effect of FKBP11 expression level on KIRC patient survival

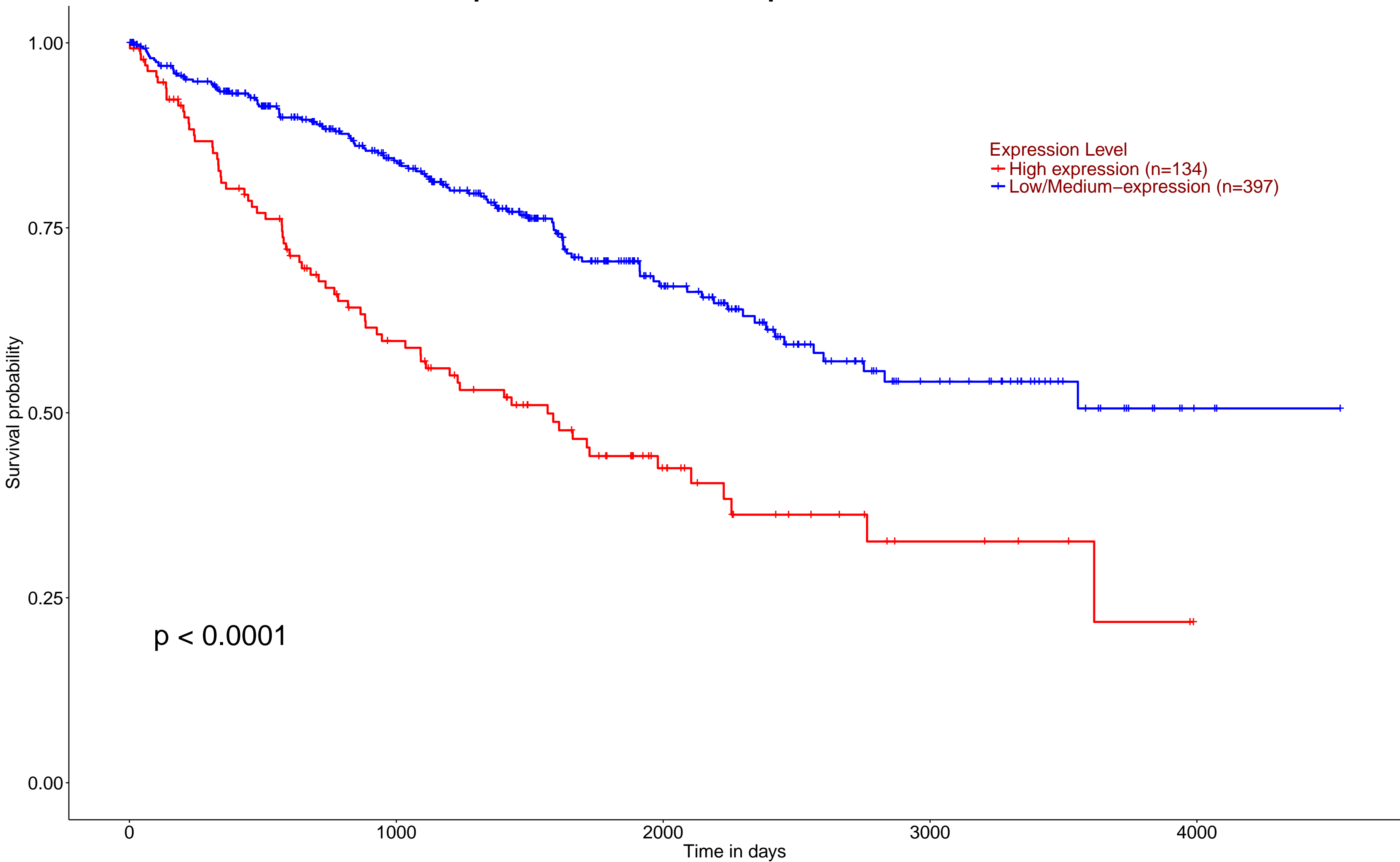

Supplement: Supplementary file 1 [file DataSheet1.zip › all raw data/Figures/Figure 6/Figure 6H.pdf]

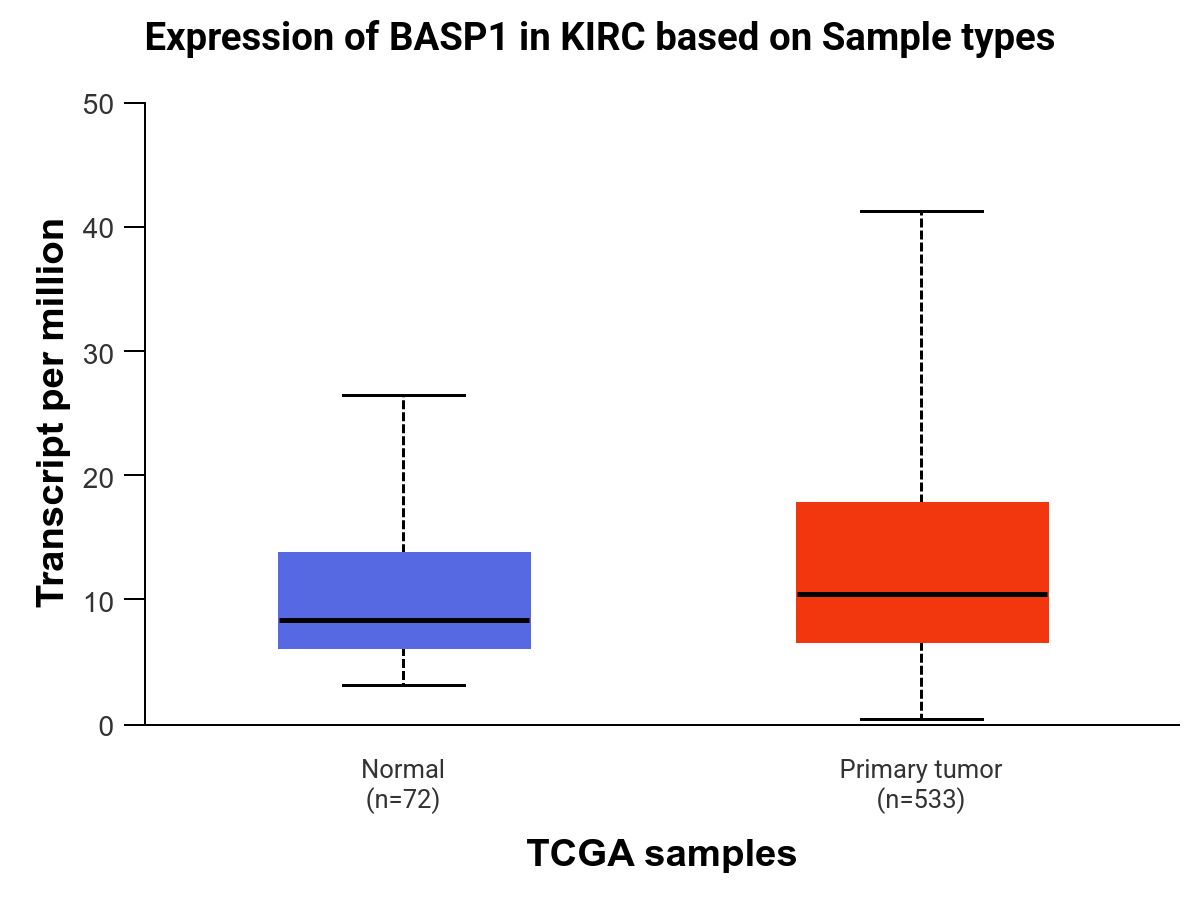

Supplement: Supplementary file 1 [file DataSheet1.zip › all raw data/Figures/Figure 7/Figure 7A.jpg]

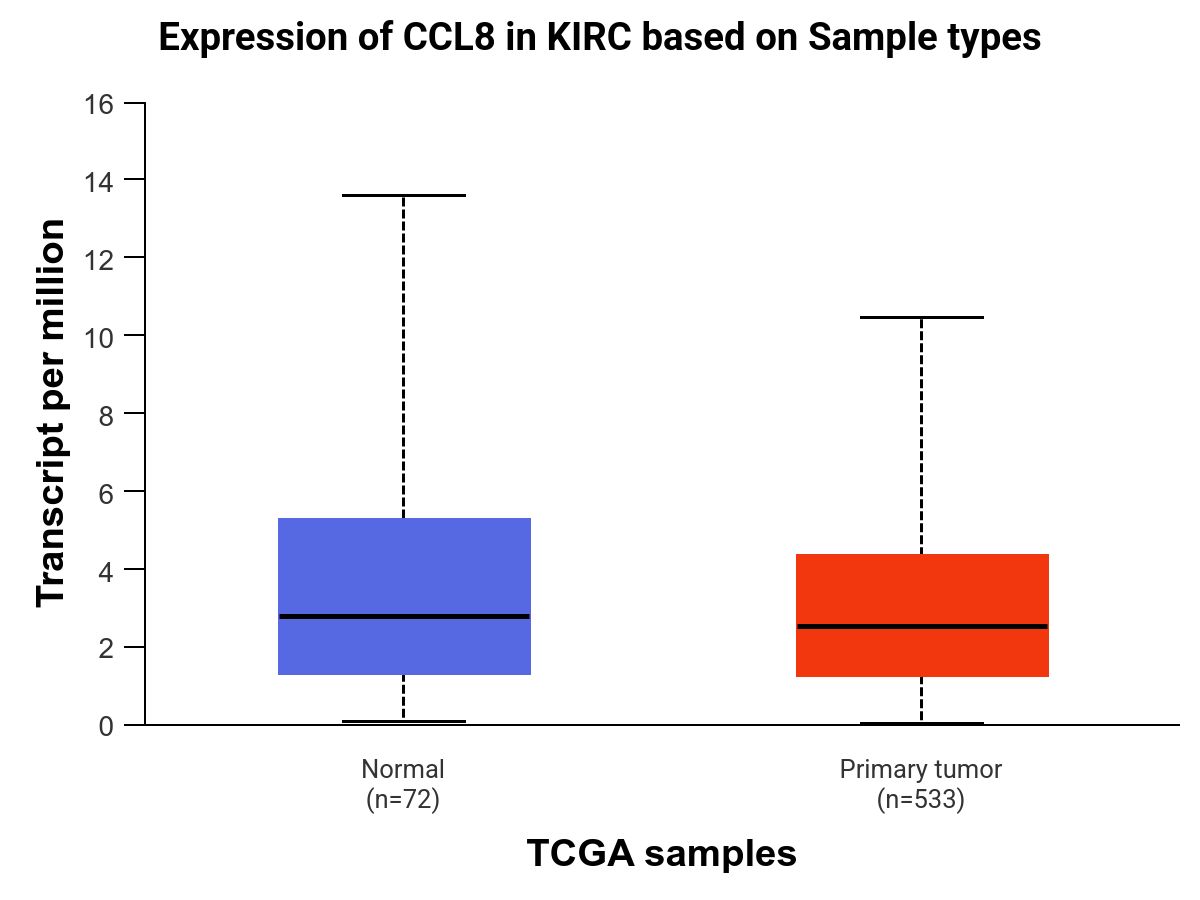

Supplement: Supplementary file 1 [file DataSheet1.zip › all raw data/Figures/Figure 7/Figure 7B.jpg]

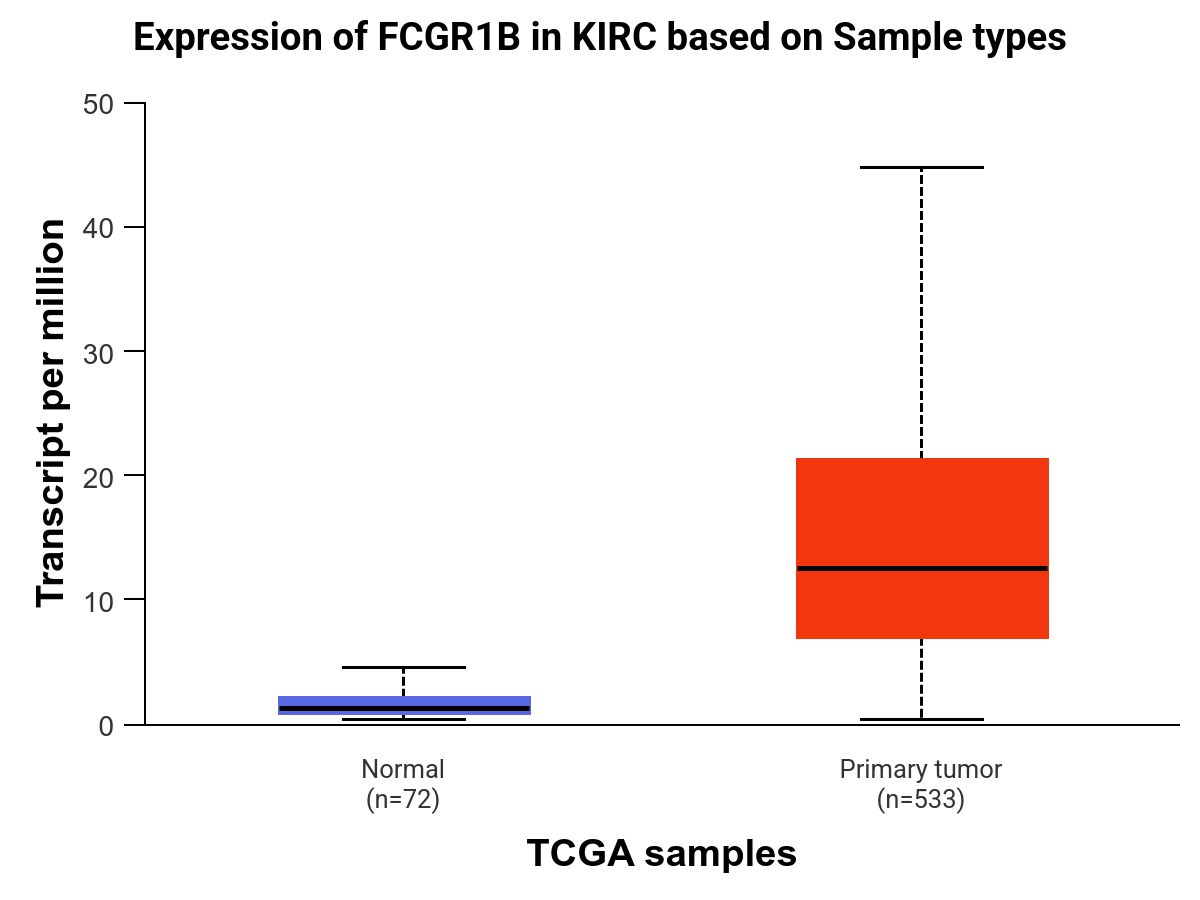

Supplement: Supplementary file 1 [file DataSheet1.zip › all raw data/Figures/Figure 7/Figure 7C.jpg]

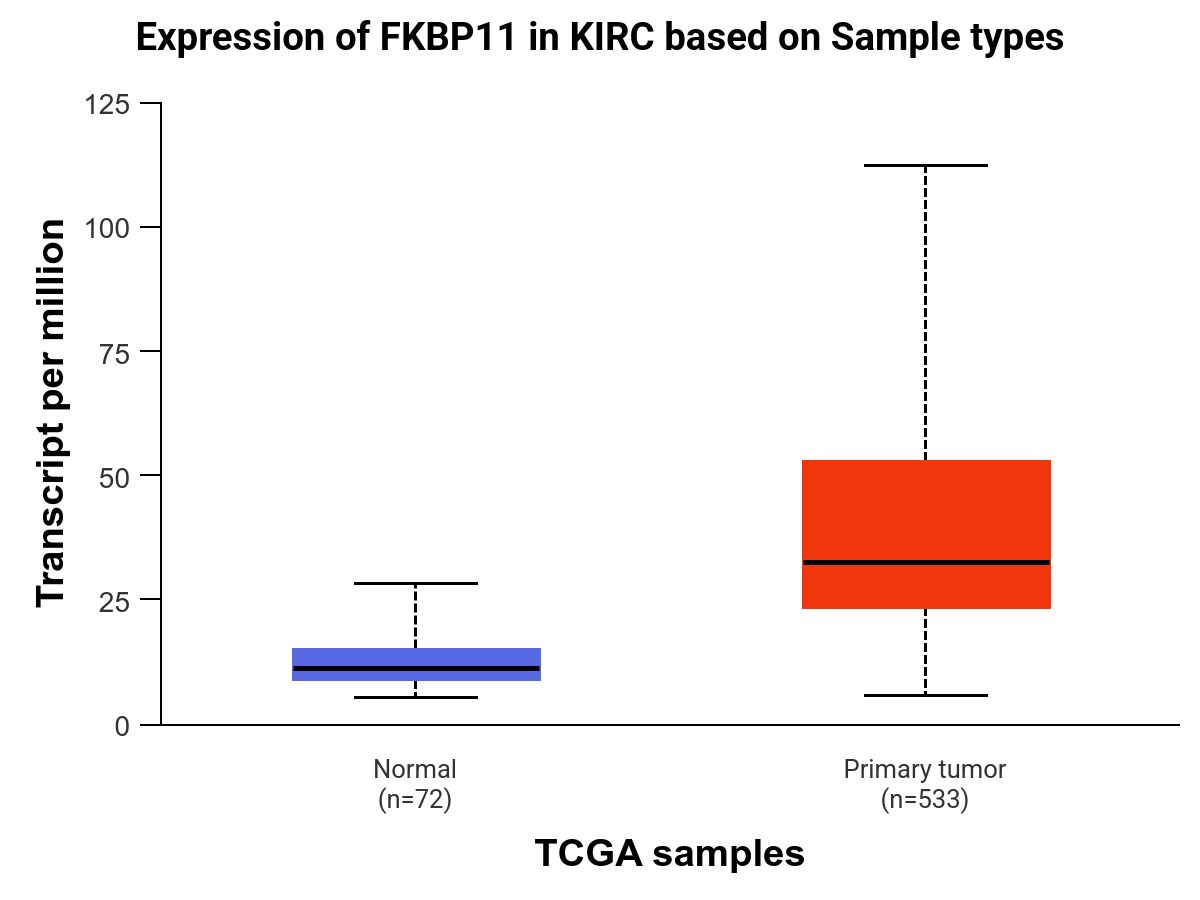

Supplement: Supplementary file 1 [file DataSheet1.zip › all raw data/Figures/Figure 7/Figure 7D.jpg]

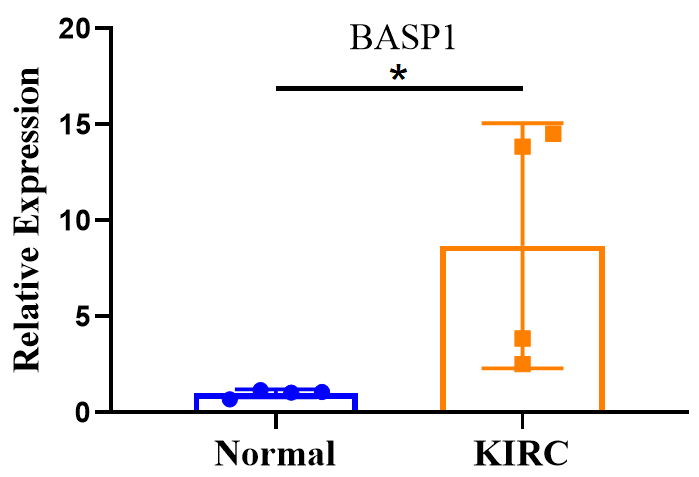

Supplement: Supplementary file 1 [file DataSheet1.zip › all raw data/Figures/Figure 7/Figure 7E.png]

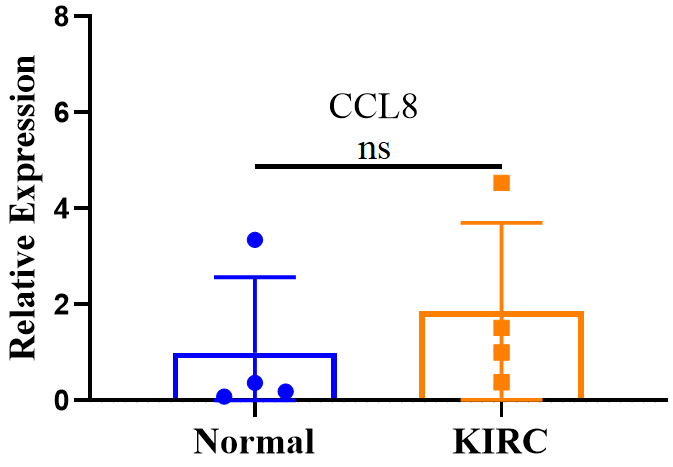

Supplement: Supplementary file 1 [file DataSheet1.zip › all raw data/Figures/Figure 7/Figure 7F.png]

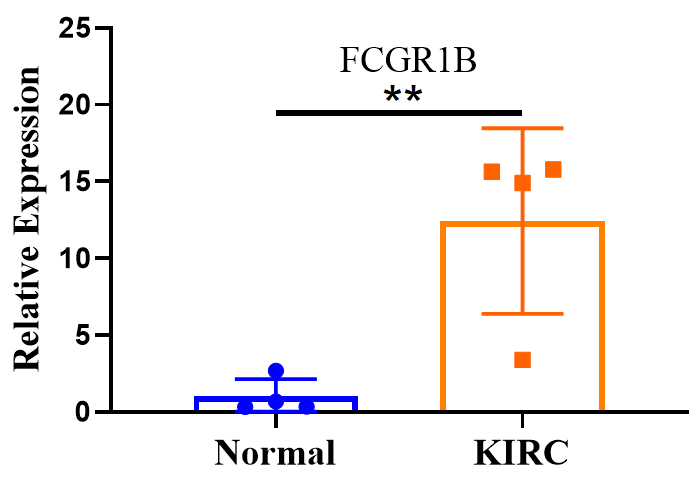

Supplement: Supplementary file 1 [file DataSheet1.zip › all raw data/Figures/Figure 7/Figure 7G.jpg]

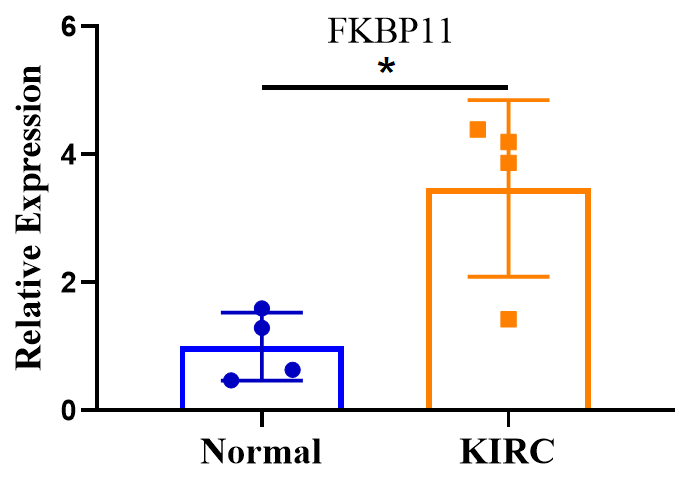

Supplement: Supplementary file 1 [file DataSheet1.zip › all raw data/Figures/Figure 7/Figure 7H.png]

Low-risk High-risk

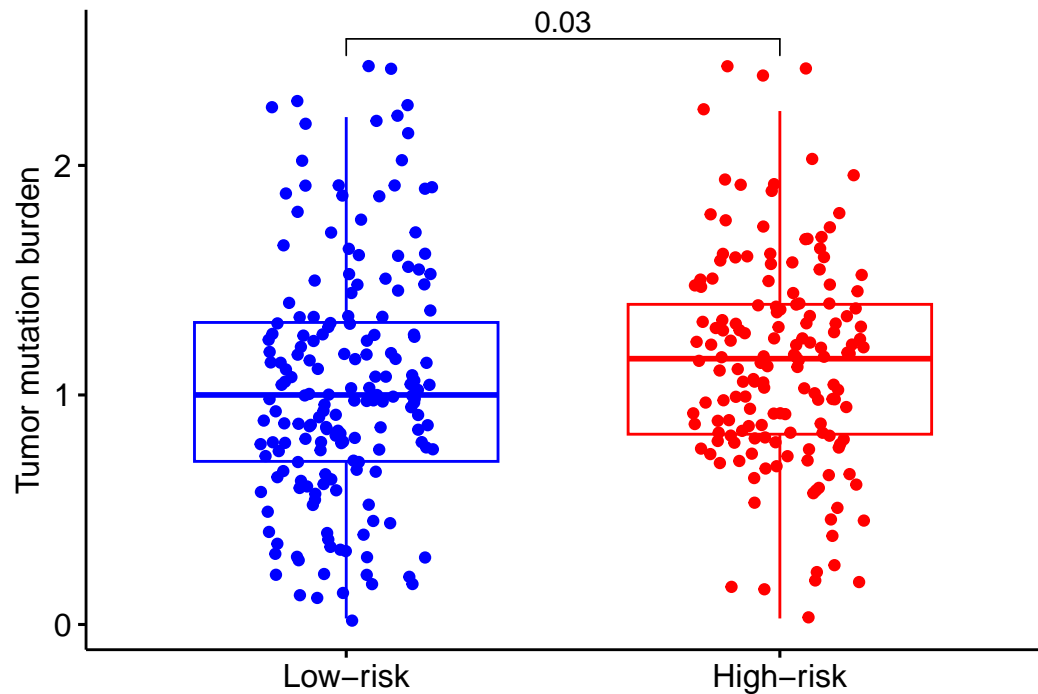

Supplement: Supplementary file 1 [file DataSheet1.zip › all raw data/Figures/Figure 8/Figure 8A.pdf]

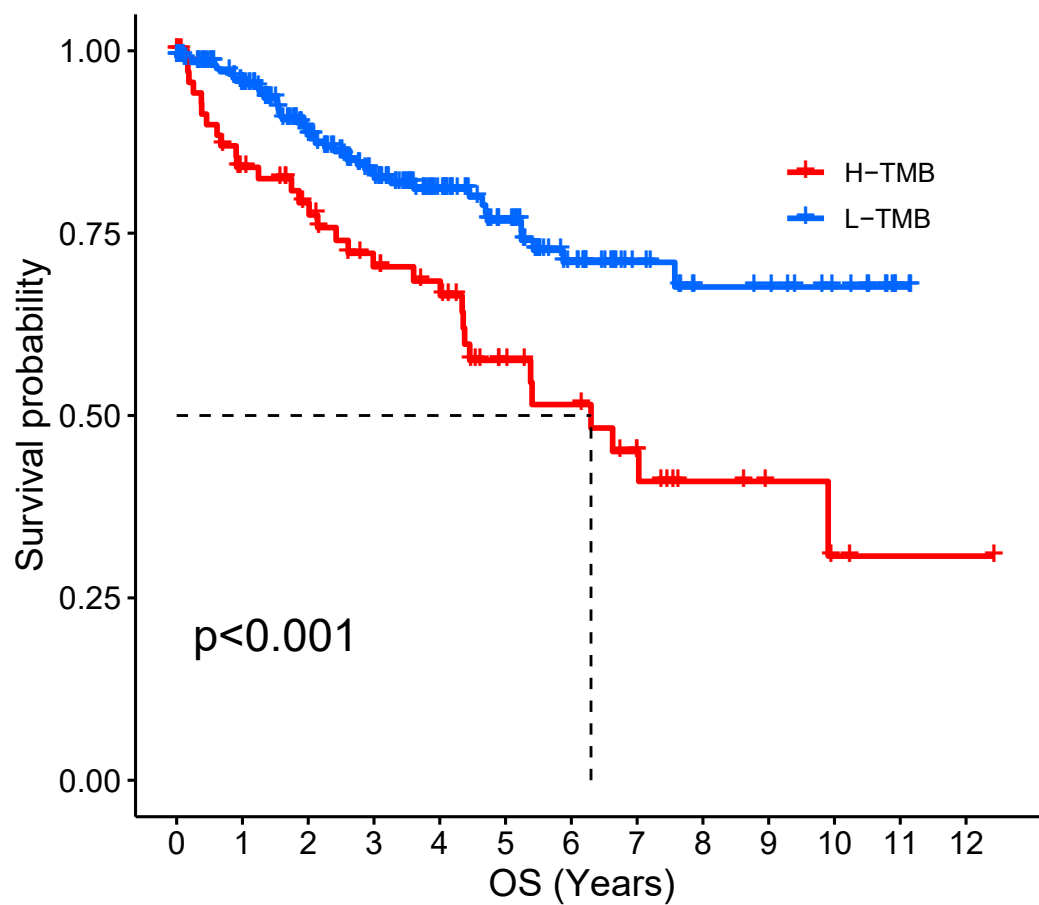

Supplement: Supplementary file 1 [file DataSheet1.zip › all raw data/Figures/Figure 8/Figure 8B.pdf]

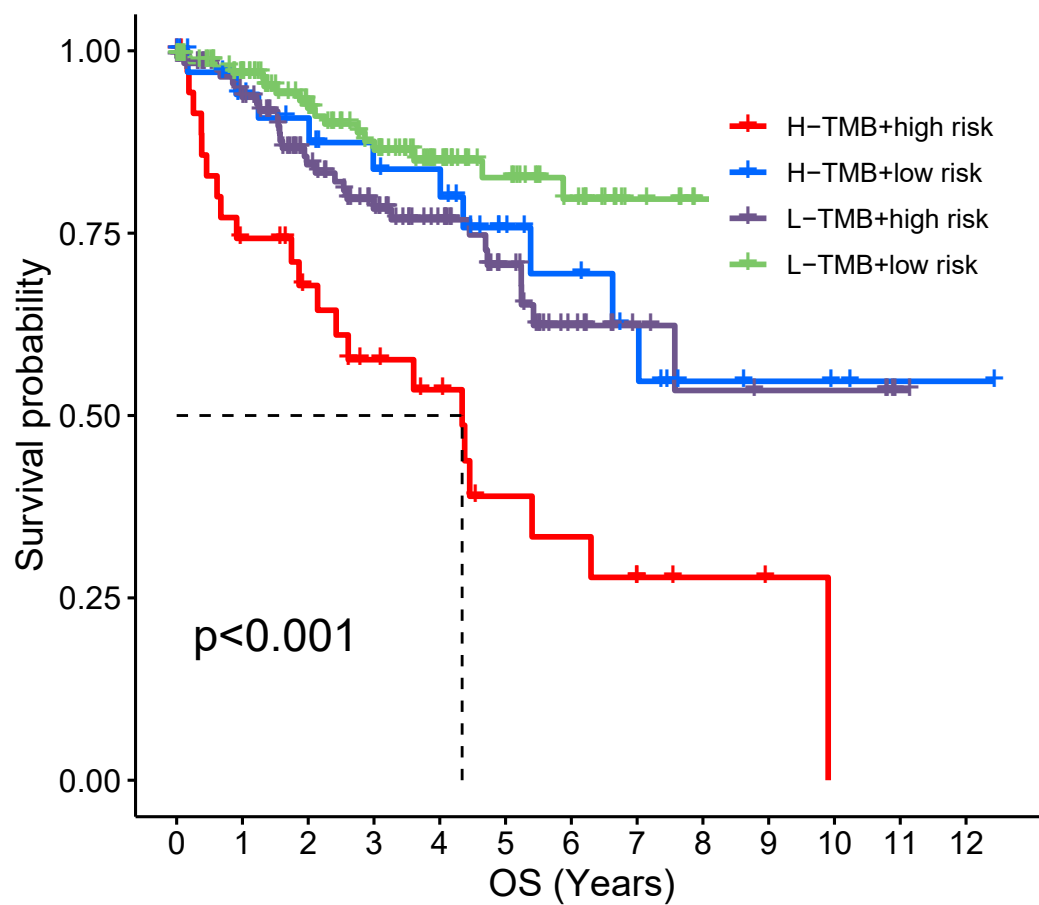

Supplement: Supplementary file 1 [file DataSheet1.zip › all raw data/Figures/Figure 8/Figure 8C.pdf]

|           | pvalue | Hazard ratio       |
|-----------|--------|--------------------|
| Age       | <0.001 | 1.032(1.018–1.045) |
| Gender    | 0.748  | 0.950(0.695–1.298) |
| Grade     | <0.001 | 2.279(1.859–2.795) |
| Stage     | <0.001 | 1.863(1.633–2.126) |
| riskScore | <0.001 | 3.121(2.364–4.121) |

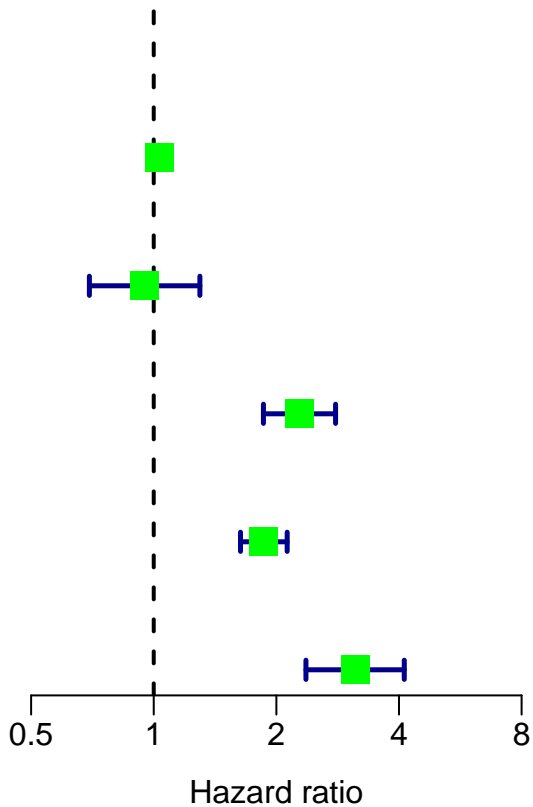

Supplement: Supplementary file 1 [file DataSheet1.zip › all raw data/Figures/Figure 9/Figure 9A.pdf]

|           | pvalue | Hazard ratio       |
|-----------|--------|--------------------|
| Age       | <0.001 | 1.035(1.020–1.050) |
| Gender    | 0.945  | 0.989(0.720–1.359) |
| Grade     | 0.011  | 1.364(1.074–1.732) |
| Stage     | <0.001 | 1.600(1.374–1.865) |
| riskScore | <0.001 | 1.836(1.291–2.610) |

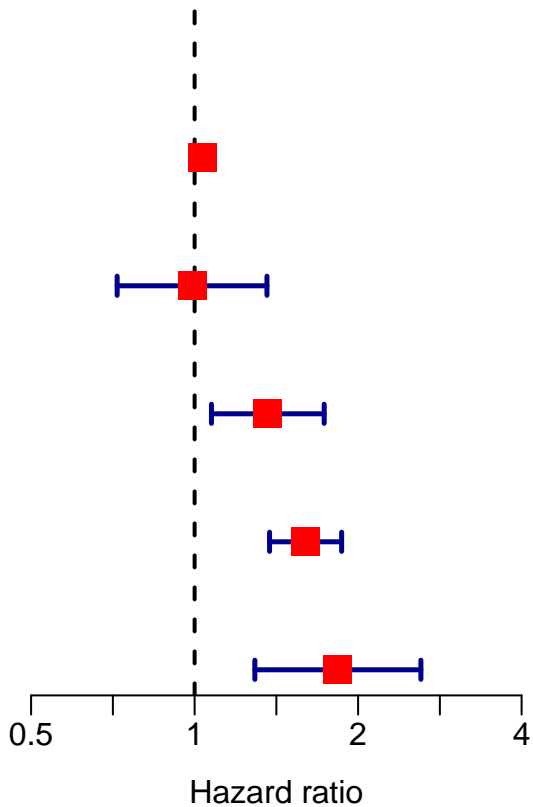

Supplement: Supplementary file 1 [file DataSheet1.zip › all raw data/Figures/Figure 9/Figure 9B.pdf]

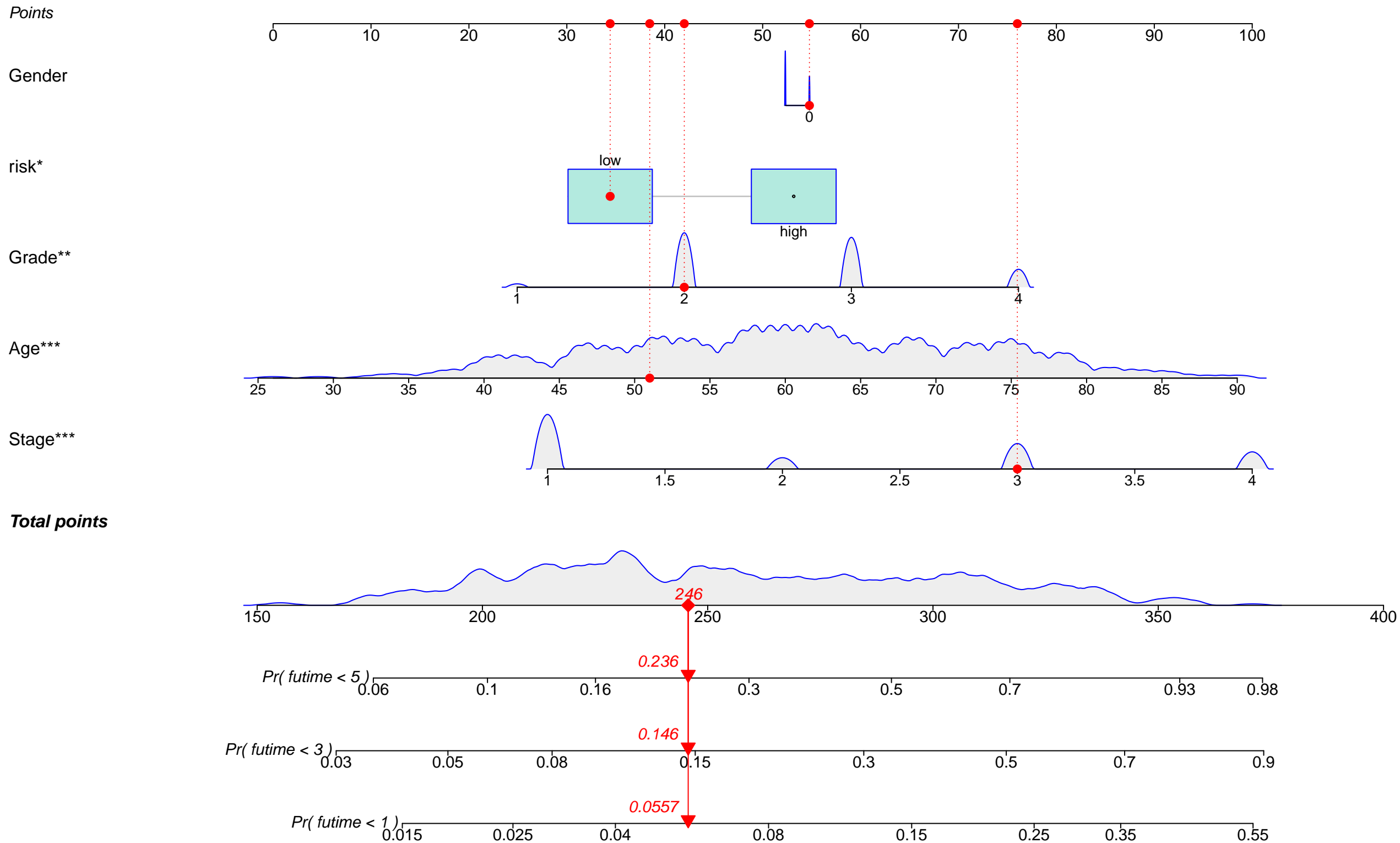

Supplement: Supplementary file 1 [file DataSheet1.zip › all raw data/Figures/Figure 9/Figure 9C.pdf]

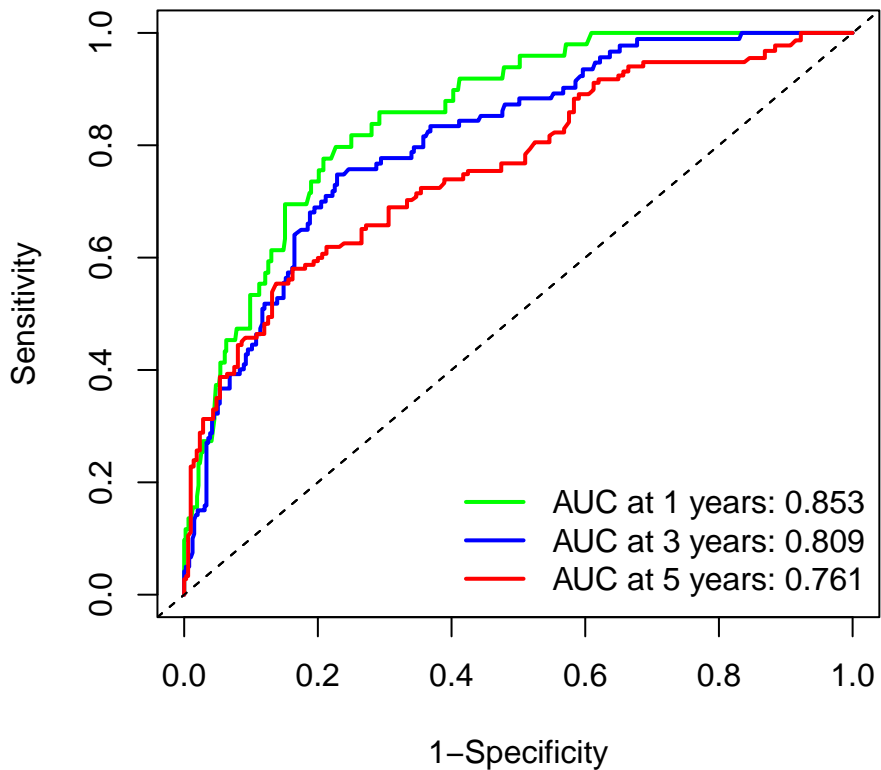

Supplement: Supplementary file 1 [file DataSheet1.zip › all raw data/Figures/Figure 9/Figure 9D.pdf]

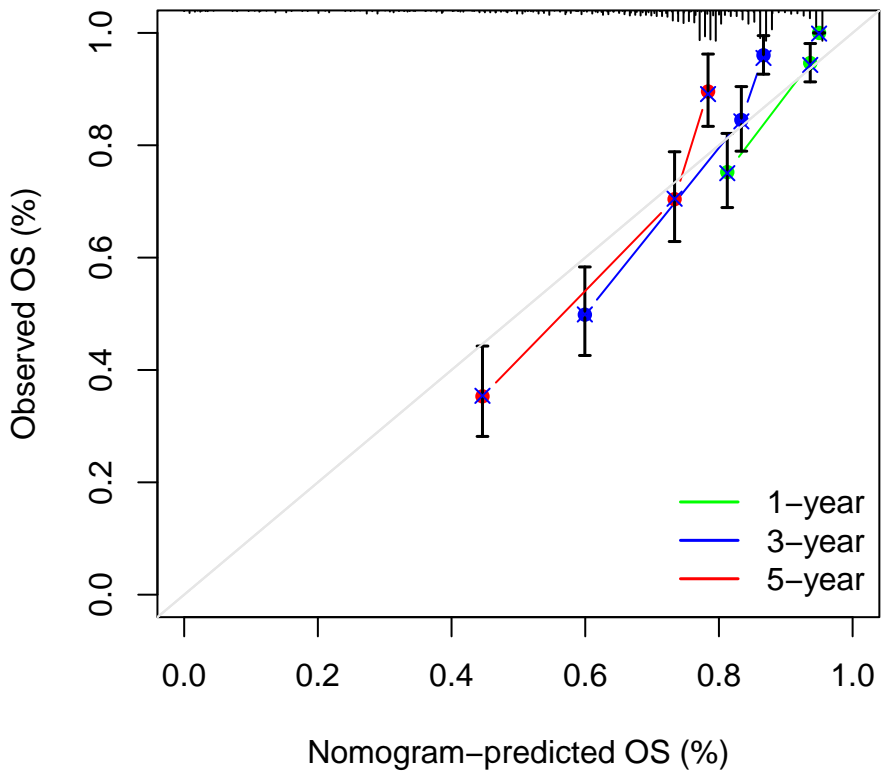

Supplement: Supplementary file 1 [file DataSheet1.zip › all raw data/Figures/Figure 9/Figure 9E.pdf]
